# A WHO digital intervention to address depression among young Chinese adults: a type 1 effectiveness-implementation randomized controlled trial

**DOI:** 10.1038/s41398-024-02812-3

**Published:** 2024-02-20

**Authors:** Gen Li, Hao Fong Sit, Wen Chen, Kunpeng Wu, Elvo Kuai Long Sou, Mek Wong, Ze Chen, Sebastian Burchert, Ieng Wai Hong, Ho Yi Sit, Agnes Iok Fong Lam, Brian J. Hall

**Affiliations:** 1 Center for Global Health Equity, New York University Shanghai, Shanghai, China; 2 Department of Psychology, The University of Hong Kong, Hong Kong SAR, China; 3 Department of Medical Statistics, School of Public Health, Sun Yat-sen University, Guangzhou, China; 4 Sun Yat-sen Centre for Migrant Health Policy, Sun Yat-sen University, Guangzhou, China; 5 Student Affairs Office, University of Macau, Macau SAR, China; 6 Centre for Macau Studies, University of Macau, Macau SAR, China; 7 Department of Education and Psychology, Division of Clinical Psychological Intervention, Freie Universität Berlin, Berlin, Germany; 8 Moon Chun Memorial College, University of Macau, Macau SAR, China; 9 Shiu Pong College, University of Macau, Macau SAR, China

**Keywords:** Depression, Human behaviour

## Abstract

Common mental disorders among young people are rising globally. Current university-based interventions are inadequate to address the need for evidence-based interventions. We investigated the effectiveness and implementation of Step-by-Step (SbS), a WHO digital intervention to address depression, among Chinese university students with depressive symptoms. In this paper, we report a type 1 hybrid effectiveness-implementation randomized controlled trial conducted between September 2021 and September 2022. The control condition was enhanced treatment as usual (ETAU, psychoeducation). The primary outcome was improvement in depression symptoms. Secondary outcomes were improvements in psychological well-being, anxiety symptoms, and self-identified psychosocial problems. Effectiveness of the intervention was evaluated using generalized linear mixed models. Implementation outcomes were evaluated by thematic analysis of participant interviews. A total of 371 participants were enrolled to two treatment conditions in a 1:1 ratio. SbS resulted in a greater reduction in depressive symptoms at posttreatment (*p* = 0.004, Hedges’ *g* = 0.35), but no significant difference between SbS and ETAU was observed at three-month follow-up (*p* = 0.179, Hedges’ *g* = 0.16). The treatment effect was larger among those who adhered to the treatment (Hedges’ *g*s = 0.59 and 0.30). Subjective well-being also improved for SbS at both time points (Hedges’ *g*s = 0.31 and 0.30). In addition, SbS resulted in more improvement in anxiety symptoms at posttreatment (*p* = 0.029, Hedges’ *g* = 0.26), but not at three-month follow-up (*p* = 0.265, Hedges’ *g* = 0.13). The qualitative results demonstrated that the intervention was well-implemented as a self-help mental health service, with minimal support from peer supporters. In conclusion, Step-by-Step, a digital intervention developed by WHO, was effective in reducing depressive symptoms in the short term and improving psychological well-being in a longer term. The sustained effect on depression needs further investigation. Improving uptake and engagement in the program is needed for its scale-up implementation as a university-based mental health service for Chinese young adults. Trial registration: ChiCTR2100050214.

## Introduction

Young people face a world with multiple challenges including political conflicts, disasters, the COVID-19 pandemic, and general uncertainty, which greatly impact their mental health [1]. The prevalence of depression in young people rose sharply in the past decade, especially among girls [2]. Evidence-based preventative and early intervention programs for depression in young people, especially in regions that lack of professional mental health workers, are in critical need [3]. Depression is a major public mental health issue among young Chinese adults. A meta-analysis of studies published between 1992 and 2020 showed that the prevalence of depression identified by self-report screening instruments was approximately 28.4% among Chinese college students [4]. Importantly, depression is associated with suicidal ideation and attempt, which is also common among Chinese young adults [5].

Despite the recognition that universities are key environments to cultivate and promote the mental well-being of young people, school-based educational interventions that focused solely on the prevention of mental disorders usually have limited effectiveness in preventing and treating depression for university students as indicated in a previous systematic review [6]. There is still a lack of evidence-based interventions to effectively address the burden of poor mental health exists in universities [7]. In addition, stigma constitutes a critical barrier that discourages university students from seeking help. Self-stigma (i.e., internalized negative self-perceptions related to mental illness) is high among Chinese students, and this is associated with lower likelihood of mental health help-seeking [8]. Cost, not knowing where to seek support, and self-reliance are other important factors that hinder young adults from seeking mental health services [9]. Finally, a lack of a trained mental health workforce that can deliver evidence-based interventions at the scale required remains a significant barrier to closing the global treatment gap. Alternative treatment approaches that can overcome this barrier are needed.

One promising strategy to deliver evidence-based interventions in university settings is the use of digital mental health interventions, defined as the use of digital technologies that help improve clients’ mental health and overall wellness [10]. In addition to overcoming barriers to care (i.e., the lack of professional service providers), digital interventions are usually more accessible without geographical limitations and lower costs [11]. It has also become a promising strategy to address mental health stigma and improve help-seeking behavior [12]. A meta-analysis of 48 interventions for university students showed the effectiveness of digital interventions in treating common mental health conditions in high-income countries [13]. However, most of the accumulating findings on digital intervention for university students are based in Western countries and populations. According to a systematic review [14], of the 89 included studies of digital interventions for depression, anxiety, and psychological well-being among college students, only 8 were from non-Western countries. As far as we know, there are few digital intervention trials designed for Chinese young adults and implemented in universities to treat depressive symptoms among college students, including the Chinese version of the *MoodGYM* intervention by Ren et al. [15], the *Best Possible Self intervention* by Auyeung & Mo [16], and the *Mindfulness for Growth and Resilience* intervention by Sun et al. [17]. All these interventions are self-help without human coaches.

Although digital mental health is fast-growing, very few of the applications are evidence-based. A review of the major smartphone app marketplaces shows that only 3.4% of apps had evidence from research to support their effectiveness, among which only 30.4% claimed to have input from mental health experts during intervention development [18]. Internet cognitive behavior therapy (iCBT) for anxiety and depression are most common digital interventions. Step-by-Step (SbS) is a behavioral activation-based digital intervention for depression designed by Carswell et al. [19]. from the World Health Organization. It is a structured minimally guided self-help intervention delivered by a smartphone application. It includes 5 story sessions which are further divided into 3 smaller parts and takes around 20 min to read. Users are recommended to complete one session every week and practice the skills and exercises that they learned from the sessions. The intervention was designed to be completed over a period of five to eight weeks. SbS is a freely available intervention and previous evidence has shown its potential to fill the gap of publicly available evidence-based psychological interventions [20]. Two recent randomized controlled trials (RCTs) conducted in Lebanon support SbS as an effective intervention for depression [21, 22]. Clinical trials showed that cultural adaptation of psychological and psychosocial interventions for depression is critical in enhancing the effectiveness of these interventions [23, 24], and a meta-analysis pointed to the increase in the effect size of adapted interventions [25]. SbS is one of the few digital mental health interventions that have been systematically culturally adapted in China. The key elements of SbS (e.g., language, metaphors, content) have undergone thorough cultural adaptation for Chinese college students [26]. Samples of major adaptations included: adding scenarios related to university life, changing characters in the narrative to improve acceptability by university students, and converting text and audio content into two different language versions (traditional Chinese and simplified Chinese). Although a single-arm feasibility study among 24 Chinese university students suggested that the adapted SbS intervention can reduce depressive symptoms at 8-week posttreatment and the intervention was safe and acceptable [27], a definitive RCT is warranted to establish intervention effectiveness.

Evaluation of intervention implementation remains unaddressed in most previous clinical trials of digital interventions, including SbS. To change practice settings to include innovation in the long term, implementation needs to be monitored and evaluated as a key component of interventions [28]. The Reach-Effectiveness-Adoption-Implementation-Maintenance (RE-AIM) framework contains five dimensions focusing on the design, dissemination, and implementation process of intervention projects, and has been most frequently used in the planning and evaluation of health projects across populations, settings and health conditions [29]. It can be applied to improve knowledge on the optimal implementation strategies and barriers to the implementation of digital interventions [30]. Following the RE-AIM framework, qualitative methods can be applied to help answer key implementation questions and inform future practices to increase the sustainability of digital interventions when scale-up is the goal.

The current study is an effectiveness-implementation randomized controlled trial conducted among students with mild or higher depressive symptoms (defined by Patient Health Questionnaire-9 score ≥5) in a university in China. The emphasis of our trial is to examine the effectiveness of SbS in reducing depressive symptoms, while concurrently evaluating the implementation of the digital intervention for Chinese young adults in university settings [31]. Based on previous trials and feasibility studies [21, 22, 27], our primary study hypothesis was that SbS would more effectively decrease depressive symptoms than the comparison condition (brief psychoeducation plus referral) at posttreatment. We also hypothesized that SbS would be more effective than comparison at three-month follow-up. Finally, we explored the effects of SbS upon symptoms of anxiety, subjective well-being, and self-defined psychological difficulties assessed by standard self-report scales at posttreatment and at three-month follow-up. Regarding implementation, we anticipated SbS would be a successful service in terms of reach, effectiveness, adoption, implementation, and maintenance.

## Methods

### Participants

The trial was introduced as a free health project only for Chinese college students in the University of Macau. Participants were recruited via (1) university daily news; (2) posters and leaflets on campus; (3) presentations by research staff; and, (4) referrals from the university counseling center from September 2021 to March 2022. Brief information about the study, QR codes, and URL of a smartphone application and a website version were provided. Upon account registration, participants received a complete description of the trial and completed informed consent. After providing electronic informed consent including privacy and data security policies of the mobile application, participants were asked to complete a brief screening test. Eligibility criteria included: being at least 18 years old, registered student in the University of Macau, Chinese citizen, or Macau resident, mild but clinically significant depressive symptoms (defined by PHQ-9 score ≥5 [32]), possessing a digital device with internet access, and native proficiency in Cantonese or Mandarin Chinese. Exclusion criteria included: (1) history of receiving regular professional psychological treatment in the past month; or, (2) report of serious thoughts or a plan to end their life in the past month. Participants excluded because of suicidal ideation were provided referrals to mental health hotlines, in-person consultation at university counseling center, and to other local clinics and hospitals. The study was approved by the Research Committee – Panel on Research Ethics of the University of Macau and the Office for Personal Data Protection, the Macau SAR Government. Participants received compensation for posttreatment and follow-up assessments ($6 and $12 US dollars, respectively).

### Procedure and randomization

A priori power analysis was conducted using two-sample t-test model by G*Power 3.1. Estimation of the effect size was based on results from previous RCT of SbS (Cohen’s *d* = 0.48 [21]). We took a more conservative estimation of Cohen’s *d* = 0.40 for sample size evaluation. According to a meta-analysis of smartphone-delivered mental health interventions, the average attrition rate at posttreatment was around 35% [33]. Therefore, we anticipated an attrition rate of 40% in our trial. A total sample size of 334 in two groups would be required to achieve a power of 80% at *p* < 0.05 for two-tailed test. A total of 689 individuals downloaded the SbS mobile App and completed the screening for study eligibility. In total, 211 participants did not meet the symptom criteria, 9 were under 18 years old, 36 had recent suicide ideation, and 62 were excluded for other reasons (e.g., recent treatment), resulting in a sample of 371 participants included in the trial. Following the screening, eligible participants were randomly assigned, by the smartphone application, to either the Step-by-Step or ETAU condition in a 1:1 ratio. A randomization sequence was generated by a permuted block randomization algorithm that was built into the app and not accessible to the research team. The algorithm generates a sequence of blocks. The length of each block is random between two and eight. Within each block, participants were assigned to the two treatment conditions with a 1:1 ratio. For those who were assigned to the SbS group, the application further downloaded full content of the SbS intervention to the smartphone and guided participants to the baseline assessment, followed by the interventional sessions. For those assigned to the ETAU group, the application only presented a brief psychoeducational and referral session to the participants after the completion of the baseline assessment.

**Fig. 1:**
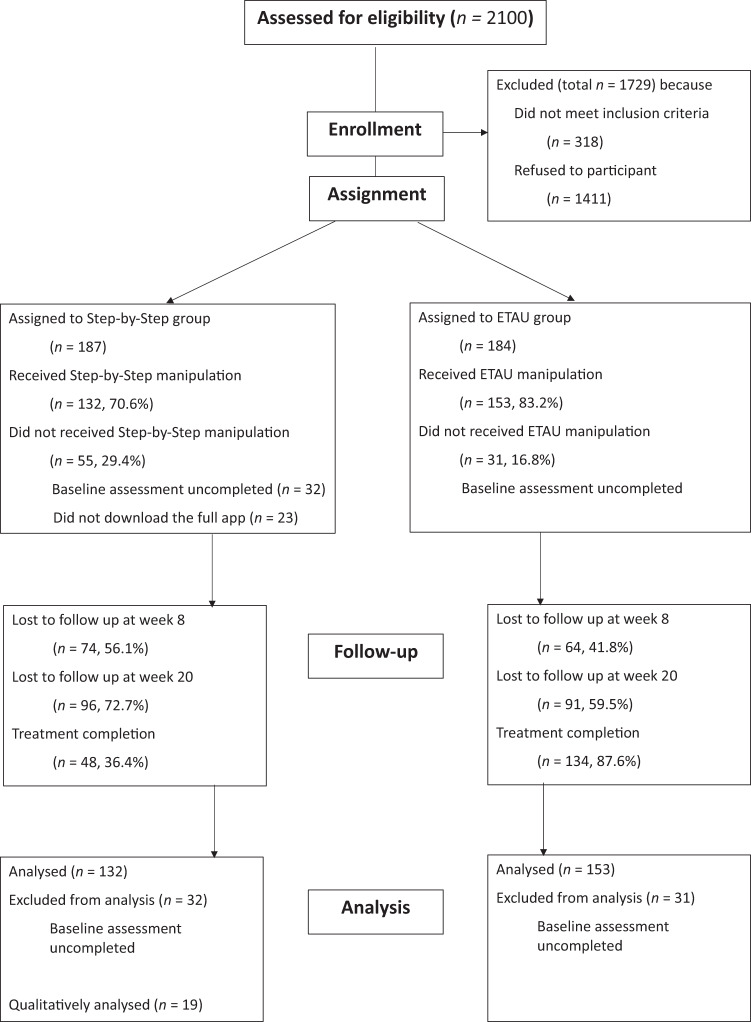
Recruitment CONSORT flowchart. Flowchart regarding the enrollment and randomization of participants.

Due to the characteristics of the psychological intervention, blinding of treatment assignment to participants was not feasible. The CONSORT flowchart of the trial is shown in Fig. 1. Of the 371 participants who were assigned to the intervention or control condition, 86 did not complete the baseline assessment or download the full mobile application, and did not enter a group, leaving a total of 132 participants in the SbS group and 153 participants in the ETAU group.

Both intervention and control conditions were delivered by the Step-by-Step Macao (“*Yibubu Aomen*”) program via a mobile application or webpage. The intervention condition was a 5-session Step-by-Step program with minimal guidance, while the control condition provided enhanced treatment as usual to participants.

### Intervention

#### Step-by-Step (SbS)

The original SbS program was developed as a transdiagnostic approach to address common mental health disorders by experts in the field of psychology, psychiatry, and global mental health, in concert with colleagues at the WHO [19]. Behavioral activation (BA) is the primary treatment component in the intervention, which is simple and effective in reducing depressive symptoms and can be delivered digitally with minimum assistance [34]. The Chinese SbS intervention was culturally adapted for Chinese university students using rigorous qualitative methods [26] and evaluated in an uncontrolled pilot trial [27].

The program content was delivered through a series of five text- and picture-based sessions that teach users knowledge about behavioral activation and interactive therapeutic practices (e.g., audio-guided relaxation exercise, plan making skills, strengthening social support and relapse prevention) that can be applied to reduce psychological distress. The sessions tell a story between a leading character who plays the role of a therapist and a student character with mental health issues. Through the story, these characters guide participants through the intervention content in a supportive and culturally appropriate manner, lead participants to try the therapeutic practices on the app and encourage more practice of these skills in their daily life. Each session lasts roughly 20–30 min, and the entire program can be completed within five weeks. The sessions teach specific techniques including: “stress management”, “planning physical and social activities”, “reducing avoidance coping”, “improving self-acceptance”, and “preventing relapse”. Participants were instructed to practice the intervention in a quiet environment and finish one session per week. Although the online sessions take about five weeks, the whole intervention took eight weeks to complete [21]. The first week was for completion of pretreatment demographic information and the second week was for the introduction session. From week 3 to week 7, participants complete the five online sessions. As the last session is for relapse prevention, participants were given another week to practice it and the posttreatment assessment was at the end of week 8.

### Control

#### Enhanced treatment as usual (ETAU)

To improve the comparability of our results with previous trials and follow the ethical principle of clinical trials, we set ETAU as the control condition. Brief psychoeducation has also been effective at reducing depressive symptoms among young adults. We thus expected modest symptom improvement within this group [35]. The ETAU included a text-only single psychoeducation session along with referrals to mental health services delivered through the mobile application. Psychoeducation provides information about how negative experiences can affect mood while referrals include a list of local primary health care centers and psychological service providers in Macao so participants can seek professional help. The psychoeducation session lasted approximately 10 min.

### Primary outcome

#### Patient Health Questionnaire-9 (PHQ-9 [36])

The PHQ-9 is a nine-item self-report scale used to assess symptoms of depression in the past two weeks. The severity of symptoms is assessed with a four-point Likert scale, ranging from 0 to 3 (0 = not at all, 1 = on several days, 2 = on more than half of the days, 3 = nearly every day), with higher scores indicating higher depressive symptoms (range: 0–27). PHQ-9 ≥ 5 is most used cutoff for mild depression, PHQ-9 ≥ 10 for moderate depression and PHQ-9 ≥ 15 for severe depression level. The PHQ-9 demonstrated good reliability and validity in the Chinese population [37, 38]. Cronbach’s α at pre-treatment was 0.74 in our sample.

### Secondary outcomes

Secondary clinical outcomes included anxiety, subjective well-being, and self-defined psychosocial problems assessed by the Generalized Anxiety Disorder-7 (GAD-7 [39]), WHO-5 Well-being Index (WHO-5 [40]) and the Psychological Outcomes Profile (PSYCHLOPS [41]), respectively. The GAD-7, WHO-5 have been validated for use among Chinese. GAD-7 and WHO-5 both demonstrated excellent internal reliability at baseline (Cronbach’s αs >0.80). Cronbach’s α for PSYCHLOPS was lower (0.67). The PSYCHLOPS has few items, and it is a secondary outcome of the intervention. Therefore, we consider the reliability of PSYCHLOPS as acceptable [42]. A more extensive description of the measures can be found in the trial protocol [43].

In consistent with previous trials of SbS, the primary outcome was measured at baseline, posttreatment (8-weeks after baseline), follow-up (3-month after posttreatment), and weekly during the intervention. Secondary outcomes were measured at baseline, posttreatment, and follow-up. Treatment satisfaction was measured by the Client Satisfaction Questionnaire for Web-Based Health Interventions (CSQ-I [44]) at posttreatment only.

### Implementation measures

The RE-AIM framework is the widest-used in public health research and the most rigorous protocol to improve the sustainability and scale-up of evidence-based practices [45]. To have a comprehensive overview of different aspects of the implementation, a RE-AIM framework-based qualitative interview guide used in the feasibility study of the SbS [27] was applied to collect data on key implementation questions among participants in the SbS group at posttreatment. The structured interview contained 21 questions regarding background information, implementation process, and SbS experience (e.g., “Was the current promotion strategies adequate for delivering SbS for students and within universities?”). Each interview was conducted by a trained interviewer and lasted for 20–30 min. After the posttreatment assessment, each participant in the SbS group was asked about their willingness to join an interview about the intervention. For those who agreed to participate, a link for a Zoom meeting was shared via email. The Zoom meetings were anonymous and recorded with the agreement of interviewees. Those who dropped out from the trial were also contacted to obtain feedback on the program and reasons for trial discontinuation.

### Treatment delivery, training, and fidelity

Student lay peer supporters recruited from two partner residential colleges of the university were trained to deliver minimal guidance to clients using Step-by-Step. All peer-supporters received training on the context and function of Step-by-Step, clinical training, as well as technical support training, and passed a performance evaluation before participating [27]. In addition, peer supporters attended weekly reflection sessions to consolidate their service skills. Each peer-supporter was responsible for 6–10 participants in total, to answer questions raised by the participants, and handle crises should they arise. For example, if they encountered self-harm or symptom deterioration, they were trained to report these events to their supervisor who could then follow-up with a treatment referral or recommend discontinuing the trial. For the participants in the SbS group, peer-supporters additionally provided anonymous periodic text messaging and weekly 15-min anonymous telephone-calls. There was no weekly contact from peer-supporters for participants in the ETAU group. Peer-supporters received weekly supervision to ensure service quality and fidelity during the intervention and to enhance their competence in providing support to participants.

### Analytic procedure

The quantitative data were analyzed using *R* by an independent biostatistician, blind to condition assignment, following the pre-registered study protocol [43]. Chi-square analyses and t-tests were performed to compare baseline characteristics between conditions and dropout status (e.g., sex, age, and educational level). We planned to follow the intention-to-treat (ITT [46]) principle for the primary analysis in the protocol. Due to unexpected high dropout at the baseline assessment and all secondary outcome measures were missing for those who did not complete the baseline, we performed a modified ITT analysis by only including participants who have finished the baseline assessment into the analysis [47]. Following the protocol, we evaluated the effectiveness of the intervention using generalized linear mixed models (GLMMs [48]). For each outcome, we conducted GLMMs which included treatment conditions (SbS vs. ETAU) and time (baseline vs. posttreatment, baseline vs. follow-up) as fixed effects, and participants as random effects. Confounders were included as fixed effects in the model and included gender, history of treatment, and history of mental illness. Hedges’ *g* and 95% confidence interval (CI) between treatment conditions were calculated for each outcome between baseline and post-intervention and baseline and 3-month follow-up for effect size estimation. The GLMMs were estimated by restricted maximum likelihood (REML) to handle missing data. As planned in the protocol, subgroup analyses were conducted by gender and baseline depression symptom severity (only for primary outcome analysis). Per protocol analyses using the same models were also conducted (PP [49]) within the sample of individuals who adhered to the treatment (i.e., 3/5 sessions of SbS; 1/1 session of psychoeducation for ETAU, *N* = 182).

Considering the high proportion of missing data, we conducted sensitivity analyses with imputed data. Although the comparisons between those who dropped out and did not drop out supported a missing at random assumption for our data, we tested the robustness of our findings to different assumptions of missing values (i.e., MAR and missing not at random, MNAR). Under the MAR assumption, we reran the GLMM models with multivariate imputation by chained equations (MICE [50]). Last observations carried forward (LOCF) is widely used under MNAR assumption and obtains conservative effect estimations of the effectiveness for interventions. Therefore, we recalculated the GLMM also using LOCF to impute missing outcome data. The detailed methodology and results for the sensitivity analyses are available in supplementary materials.

For qualitative data, each audio record of the interviews was transcribed verbatim. A deductive thematic analysis framework was used for the content analysis. After quality control of the transcriptions, an initial codebook was developed based on the reach, effectiveness, adoption, implementation, and maintenance questions of the current intervention according to the transcripts. The transcripts were then coded based on the codebook independently by two research staff who were familiar with the interview guideline. Any inconsistency was solved through discussion. The codebook is attached as a supplementary material for the paper.

## Results

### Sample characteristics

Means and SDs of the demographic and clinical outcome data are presented in Table 1. Most participants were female (69.1%), undergraduate students (68.1%), and were from mainland China (67.4%). Chi-squared and t-tests showed no baseline treatment condition differences in gender, age, or other demographics. There was no difference in baseline primary or secondary outcomes except for well-being, which was slightly higher in the SbS group compared to the ETAU group (*t*_283_ = 2.16, *p* = 0.032).

**Table 1:** Pretreatment demographic and clinical characteristics of intent-to-treat sample receiving Step by Step (SbS) and enhanced treatment as usual (ETAU).

	Overall (*N* = 285)	Step by Step (*N* = 132)	ETAU (*N* = 153)	*p*	Posttreatment assessment complete (*N* = 147)	Posttreatment assessment incomplete (*N* = 138)	*p*
Age (mean (SD))	20.84 (3.27)	20.89 (3.37)	20.79 (3.18)	0.797	20.85 (2.97)	20.83 (3.56)	0.967
Gender (%)				0.947			0.077
Male	88 (30.9)	40 (30.3)	48 (31.4)		38 (25.9)	50 (36.2)	
Female	197 (69.1)	92 (69.7)	105 (68.6)		109 (74.1)	88 (63.8)	
Education status (%)				0.554			0.332
Completed high school	12 (4.2)	7 (5.3)	5 (3.3)		4 (2.7)	8 (5.8)	
Undergraduate student	194 (68.1)	90 (68.2)	104 (68.0)		44 (29.9)	34 (24.6)	
Finished Undergraduate or above	78 (27.4)	34 (25.8)	44 (28.8)		99 (67.3)	95 (68.8)	
Born in Macao (%)				0.552			0.592
No	192 (67.4)	87 (65.9)	105 (68.6)		100 (68.0)	92 (66.7)	
Yes	92 (32.3)	45 (34.1)	47 (30.7)		46 (31.3)	46 (33.3)	
Source of referral to trial (%)				0.82			0.643
Resident college mentor	99 (34.7)	46 (34.8)	53 (34.6)		53 (36.1)	46 (33.3)	
Website	54 (18.9)	27 (20.5)	27 (17.6)		31 (21.1)	23 (16.7)	
Counseling center/social worker	35 (12.3)	18 (13.6)	17 (11.1)		18 (12.2)	17 (12.3)	
Friends	10 (3.5)	3 (2.3)	7 (4.6)		5 (3.4)	5 (3.6)	
Student societies/NGO	5 (1.8)	2 (1.5)	3 (2.0)		1 (0.7)	4 (2.9)	
Other	57 (20.0)	27 (20.5)	30 (19.6)		29 (19.7)	28 (20.3)	
History of mental disorder diagnosis in the past 3 months (%)				0.88			0.57
No	281 (98.6)	130 (98.5)	151 (98.7)		146 (99.3)	135 (97.8)	
Yes	4 (1.4)	2 (1.5)	2 (1.3)		1 (0.7)	3 (2.2)	
History of pharmacotherapy in the past 3 months (%)				0.513			0.57
No	281 (98.6)	129 (97.7)	152 (99.3)		146 (99.3)	135 (97.8)	
Yes	4 (1.4)	3 (2.3)	1 (0.7)		1 (0.7)	3 (2.2)	
History of psychotherapy in the past 3 months (%)				0.513			0.66
No	281 (98.6)	129 (97.7)	152 (99.3)		144 (98.0)	137 (99.3)	
Yes	4 (1.4)	3 (2.3)	1 (0.7)		3 (2.0)	1 (0.7)	
PHQ-9 score (SD)	10.45 (4.32)	10.12 (4.07)	10.73 (4.52)	0.24	10.31 (4.12)	10.59 (4.54)	0.593
WHO-5 score (SD)	11.18 (4.51)	11.80 (4.30)	10.65 (4.62)	**0.032**	11.36 (4.50)	10.99 (4.52)	0.484
GAD-7 score (SD)	8.33 (4.48)	8.08 (4.34)	8.54 (4.61)	0.39	8.33 (4.56)	8.33 (4.42)	0.99
PSYCHLOPS score (SD)	12.50 (3.54)	12.30 (3.62)	12.67 (3.47)	0.399	12.46 (3.65)	12.53 (3.43)	0.875

Missing responses (3 for age, one for education, one for place of born, 25 for source of referral to trial, and 25 for self-defined psychosocial problems)

### Primary outcome

Consistent with our hypotheses, the GLMM analyses of the modified ITT sample (Table 2) showed that improvements in depressive symptoms were greater in the SbS group when compared with the ETAU group (*p* = 0.004, Hedges’ *g* = 0.35, 95% CI: 0.12–0.59). The difference in the treatment effect between groups did not remain significant at 3-month follow-up (*p* = 0.179, Hedges’ *g* = 0.16, 95% CI: −0.07–0.40). The subgroup analyses indicated that the effectiveness of SbS was greater among female participants (*p* = 0.025, Hedges’ *g* = 0.32, 95% CI: 0.04–0.60) at posttreatment (Table 3). The subgroup analysis also implied that those with mild and severe depressive levels at baseline (Hedges’ *gs*: 0.46–0.52) would benefit more from the SbS treatment than those with moderate severity (Hedges’ *g* = 0.27) at posttreatment. Per-protocol analyses showed that, the effect size of the SbS intervention increased to moderate at posttest (*p* < 0.001, Hedges’ *g* = 0.59, 95% CI: 0.25–0.93) if the participant adhered to the treatment (Table 4). In PP analysis, there was still a lack of evidence of the long-term effectiveness of SbS over ETAU (*p* = 0.086, Hedges’ *g* = 0.30, 95% CI: −0.03–0.64). The sensitivity analyses using MICE for missing data imputation replicated all the findings in the primary analysis while the results of LOCF also demonstrated better effectiveness of SbS than ETAU at 3-month follow-up (see Supplementary Materials).

**Table 2:** Post-treatment and long-term effectiveness of Step by Step in comparison to enhanced treatment as usual in the modified intent-to-treat sample.

	Modified ITT sample (*n* = 285)					
	Posttreatment			Follow-up		
	*β* (95% CI)	*p*	Hedges’ *g* (95% CI)	*β* (95% CI)	*p*	Hedges’ *g* (95% CI)
Primary outcome						
PHQ-9	1.43 (0.47, 2.39)	**0.004**	0.35 (0.12, 0.59)	0.66 (−0.30, 1.61)	0.179	0.16 (−0.07, 0.40)
Secondary outcomes						
WHO-5	−1.35 (−2.36, −0.33)	**0.01**	0.31 (0.08, 0.54)	−1.33 (−2.35, −0.31)	**0.011**	0.30 (0.07, 0.54)
GAD-7	1.12 (0.12, 2.13)	**0.029**	0.26 (0.03, 0.49)	0.55 (−0.42, 1.52)	0.265	0.13 (−0.10, 0.37)
PSYCHLOPS	0.72 (−0.10, 1.54)	0.085	0.21 (−0.02, 0.45)	0.58 (−0.25, 1.41)	0.173	0.17 (−0.06, 0.41)

GLMM was employed with adjusted covariates including time, participants, gender, history of past treatment, and history of mental illness.

Bold values identify statistical significance (*p* < 0.05).

**Table 3:** Subgroup analyses of the effectiveness of Step by Step in comparison to enhanced treatment as usual in treating depression symptoms.

	Subgroups	Modified ITT sample (*N* = 285)		
		*β* (95% CI)	*p*	Hedges’ *g* (95% CI)
Posttreatment				
	Gender^a^			
	Female (*n* = 197)	1.30 (0.17, 2.43)	**0.025**	0.32 (0.04, 0.60)
	Male (*n* = 88)	1.53 (−0.28, 3.34)	0.101	0.40 (−0.03, 0.82)
	Depression severity^b^			
	Mild (*n* = 140)	1.00 (0.28, 1.71)	**0.007**	0.45 (0.11, 0.78)
	Moderate (*n* = 97)	0.77 (−0.17, 1.70)	0.111	0.27 (−0.13, 0.68)
	Severe (*n* = 48)	2.00 (−0.20, 4.19)	0.081	0.52 (−0.06, 1.10)
Follow-up				
	Gender^a^			
	Female (*n* = 197)	0.58 (−0.48, 1.64)	0.282	0.16 (−0.13, 0.44)
	Male (*n* = 88)	0.74 (−1.28, 2.76)	0.476	0.16 (−0.26, 0.58)
	Depression severity^b^			
	Mild (*n* = 140)	0.13 (−0.44, 0.71)	0.647	0.07 (−0.26, 0.41)
	Moderate (*n* = 97)	−0.52 (−1.38, 0.33)	0.234	−0.22 (−0.62, 0.19)
	Severe (*n* = 48)	1.72 (−0.33, 3.78)	0.107	0.47 (−0.12, 1.05)

^a^Adjusted covariates included time, participants, history of past treatment, and history of mental illness.

^b^Adjusted covariates included time, participants, gender, history of past treatment, and history of mental illness.

**Table 4:** Post-treatment and long-term effectiveness of Step by Step in comparison to enhanced treatment as usual in the per-protocol sample.

	Per-protocol sample (*n* = 182)					
	Posttreatment			Follow-up		
	*β* (95% CI)	*p*	Hedges’ *g* (95% CI)	*β* (95% CI)	*p*	Hedges’ *g* (95% CI)
Primary outcome						
PHQ-9	2.14 (0.89, 3.38)	**0.001**	0.59 (0.25, 0.92)	1.09 (−0.15, 2.32)	0.086	0.30 (−0.03, 0.64)
Secondary outcomes						
WHO-5	−1.56 (−2.91, −0.21)	**0.025**	0.40 (0.06, 0.73)	−1.83 (−3.17, −0.48)	**0.009**	0.46 (0.13, 0.79)
GAD-7	1.02 (−0.32, 2.37)	0.138	0.26 (−0.08, 0.59)	0.28 (−0.96, 1.53)	0.658	0.08 (−0.25, 0.41)
PSYCHLOPS	0.96 (−0.11, 2.04)	0.08	0.31 (−0.02, 0.64)	1.09 (0.03, 2.15)	**0.045**	0.37 (0.04, 0.70)

GLMM was employed with adjusted covariates including time, participants, gender, history of past treatment, and history of mental illness.

### Secondary outcomes

Consistent with expectations, the modified ITT analyses showed that SbS is more effective in improving psychological well-being when compared with ETAU (*p* = 0.01, Hedges’ *g* = 0.31, 95% CI: 0.08–0.54), and the effect remained at 3-month follow-up (*p* = 0.01, Hedges’ *g* = 0.30, 95% CI: 0.07–0.54). Subgroup analyses indicated that the improvements in well-being were only observed among males at both time points (see Supplementary Materials). Modified ITT indicated that SbS had a small and significant effect in treating anxiety symptoms at posttreatment (*p* = 0.029, Hedges’ *g* = 0.26, 95% CI: 0.03–0.49), which was non-significant at follow-up (*p* = 0.265, Hedges’ *g* = 0.13, 95% CI: −0.10–0.37). Finally, per-protocol analyses suggested that SbS reduced self-defined psychosocial problems at 3-month follow-up (Hedges’ *g* = 0.37). The sensitivity analyses using LOCF and MICE had consistent results with the main findings (see Supplementary Materials).

### Treatment adherence, satisfaction, utilization of additional treatment, and dropout

No adverse events were reported during the trial. Depressive symptom deterioration in the intervention group was assessed as defined by Katon et al. [51]. (1) PHQ-9 ≥ 10 at posttreatment; (2) at least two symptoms worsening from pre- to post-treatment. Two participants in the SbS group met these criteria for symptom deterioration. Neither of them endorsed self-harm/suicide symptom at posttreatment.

Treatment adherence was defined as over half of the treatment sessions completed (3 of 5 sessions for SbS, consistent with previous feasibility studies of SbS [27, 52]. The average number of sessions completed was 1.9 (SD = 2.1) in the SbS group. Of the five interventional sessions, over half (53.8%) of the participants completed at least one, and about one in four (24.2%) completed all five sessions.

Treatment satisfaction measured by CSQ-I at posttreatment was significant higher in the SbS group (mean = 26.2, *SD* = 4.6) than in the ETAU (mean = 22.7, *SD* = 4.9) group (*t*_137_ = 4.19, *p* < 0.001). There was no significant difference in treatment satisfaction between those who adhered (mean = 24.0, *SD* = 5.1) and did not adhere (mean = 25.0, *SD* = 5.2) to their assigned treatment. Three participants in the SbS group and four in the ETAU group reported receiving psychopharmacotherapy, and one participant in the SbS group reported receiving psychotherapy during the intervention.

The dropout rate was high for both conditions. The dropout rates at post-assessment were 56.1% for SbS and 41.8% for ETAU. Regarding the three-month follow-up, the rates were 72.7% and 59.5%, respectively. Chi-square tests showed the dropout rates were higher in the SbS group at both time points (*p*s = 0.017 and 0.019, respectively). None of the demographics and clinical outcomes at baseline was predictive of the dropout status at posttreatment (see Table 1). Of those who dropped out from the SbS condition, 77.0% were *early dropouts* (defined as dropout before completion of intervention session 1 [53]. A total of 62 participants in the SbS group reported their reasons for dropout: 29.0% did not establish contact with their peer-supporter, 25.8% thought the content was too much for them and had no time to finish, and 21.0% thought they did not really need the service and it was therefore not helpful. Other reasons for dropout included leaving the campus, not liking the app design, and technology issues with the software.

### Implementation outcomes

Invitation to a semi-structured interviews were sent to participants in the SbS group who completed the posttreatment assessment. Nineteen participants accepted the invitation. No significant differences were observed between those who accepted or did not accept the interview in terms of baseline or posttreatment PHQ-9 score (*p* = 0.937 and 0.486). The responses to these interviews were organized according to the codebook.

### Reach

Feedback was collected on the SbS recruitment channels. Most participants were linked to the trial via university news emails, posters, and leaflets posted on the campus, and some via presentations given by research staff. Over half of the interviewees considered current recruitment advertisements as inadequate to reach the target population as emails or posters can easily be ignored and the introduction failed to attract people with less mental health awareness. Suggestions to enhance reach included social media communities such as *WeChat* or *Instagram*. For offline promotion, the most mentioned suggestions were posters or on-campus activities. The most recommended promotion strategy was to provide more targeted information (e.g., potential benefits, who will benefit) in a more eye-catching way. The potential of the intervention to address mental health conditions commonly experienced by college students such as academic stress and procrastination can be highlighted.

### Effectiveness

On a scale from one to ten, participants reported an average of seven regarding the effectiveness of the intervention to reduce their stress. Those who gave lower rating mentioned that they did not really have stress issues. Most participants found the SbS had more content and was more well-structured than their expectation before downloading. They reported that it was like a toolbox to help relieve stress rather than simply a list of information, and psychological assessments. The intervention matched the expectation of most clients except two who thought it took too long to unlock new sessions and features, and therefore, people in acute distress may be missed. Over half of the clients reported they learned new knowledge and skills on how to regulate mood, make plans, and get rid of negative moods.

### Adoption

We evaluated to what extent the different aspects of the digital intervention were adopted by the clients. Feedback on the length and content of assessments in the app was all positive, expect one report of the inconvenience to skip specific items. The language and the stories in different sessions were easy to understand in a Chinese context. The interactive online activity aiming to activate the clients by making a plan for a challenging activity and voice-guided ground exercise or breathing training were the most adopted features of the app. Most clients applied the skills learned from the intervention in their day-to-day life. The behavior activation modules are most often used to list out plans for daily activities and set milestones to achieve a goal. The breathing practice was also commonly used, to help deal with sleep disturbances and relieve anxiety.

### Implementation

The most mentioned problem encountered by the clients was forgetting to use the program, as the interval between sessions (i.e., one week) was too long for them. Because lack of supervision and motivation, some participants found it hard to put the behavior activation plans into practice. These barriers suggest that more involvement of peer supporters to increase treatment fidelity is critical for the successful implementation of the intervention. Sixteen of the interviewed participants established regular contact with their peer supporters. Most of them reported positive qualities about their supporters, including patience, kindness, being good at reflective listening and giving positive feedback to improve engagement. Almost everyone reported their supporter was helpful in providing technical guidance, including content-based feedback and troubleshooting, along with providing reminders and encouragement to continue the intervention. Despite the positives of having a peer-supporter, two participants suggested that weekly contact was too frequent, and three participants received only one call during the trial.

### Maintenance

The students were asked what improvements are needed for long-term and wider implementation of the intervention. Some provided suggestions from a technical perspective. Many suggested to switch to other delivery channels, such as a *WeChat* mini program, which requires less storage, allows more reminders, and may have better stability. Others gave suggestions on the content. In general, more multimedia content with regular updating is needed. The stories can be extended to more chapters, with different stressful settings students may encounter in their campus life (e.g., academic stress, peer pressure, bullying). More interactive features can be integrated into the stories. Another point often mentioned was to provide customized content for clients with different symptom severity, especially for those with mild symptoms.

## Discussion

In this study, we described a type 1 hybrid randomized controlled trial for a digital mental health intervention (SbS) among Chinese young adults. Consistent with the study hypotheses, SbS was more effective than ETAU in reducing depressive symptoms regardless of treatment adherence. The between group treatment effect did not remain significant at three-month follow-up. Per-protocol analysis revealed that if participants completed over half of the sessions, the effect size between conditions were moderate at posttreatment. The intervention also improved subjective well-being, especially among young males. The intervention also helped solve self-identified psychosocial difficulties among participants adhering to the intervention. The overall feedback on the intervention implementation in a university setting was positive, but the relatively low engagement remained a major barrier for implementation. Further modifications needed before scaling up the intervention to other Chinese-speaking young adults were identified, in terms of promotion strategies, delivery channels, and intervention content. The results support SbS as an evidenced-based mental health intervention with potential to be adopted by universities to improve mental well-being of young adults in China. Our findings also pointed to some key strategies that can be applied to improve treatment fidelity in future implementation.

The main findings regarding the effectiveness of SbS in this trial were generally consistent with the previous feasibility study among Chinese youth [27], and randomized controlled trials among residents and refugees in Lebanon [21, 22]. In each trial, a greater reduction in depressive symptoms was noted in SbS than enhanced treatment as usual (effect sizes from 0.48 to 0.71). Together these results demonstrate that SbS is an effective intervention to reduce depressive symptoms in several populations and geographic locations where professional mental health service are limited (there are roughly 1.7 PhD-level clinical psychologists or psychiatrists per 100,000 adults in the Chinese speaking population in Macao according to our preliminary desk reviews and interviews with local stakeholders [54]). The current study also extends previous trial results and is the first to show that SbS can address mild depression symptoms. Progress in psychopathology studies has shown that mental disorders like depression exist on a severity spectrum rather than exist as categories [7, 55] Mild, or sub-clinical depression is known to cause significant functional impairment [56] and may develop into a depressive disorder without appropriate treatment [57]. Although SbS was designed as a transdiagnostic intervention for those with more severe depressive conditions, results from our subgroup analyses also provide the first evidence to support SbS as an effective treatment for less severe depression and support its potential use as a preventative intervention. Accordingly, the small effect size observed in the current trial compared to previous trials with moderate effect sizes and the insignificant long-term effects could be due to the floor effect caused by a lower baseline depressive symptom in our trial. An investigation of SbS on long-term mental health (e.g., symptoms or profile) among young adults with mild symptoms would provide further evidence of its effectiveness as a prevention program.

Partly consistent with our hypothesis, the SbS has a short-term effectiveness in improving anxiety symptoms, but the effect size was smaller than depressive symptoms. The long-term effectiveness on anxiety was also nonsignificant. We did not find any evidence supporting the effectiveness of SbS in solving self-defined psychosocial problems. SbS was initially developed as a guided online self-help version of Problem Management Plus (PM+), another WHO transdiagnostic intervention for adults suffering from mental distress and self-identified practical problems [58]. However, because the problem management component of PM+ could not be easily adapted for the online version, SbS specifically focuses on behavioral activation to reduce depressive symptoms and thus was less transdiagnostic than it was intended to be [59]. This might explain why SbS was less effective in reducing anxiety symptoms than depression symptoms and did not reduce self-defined psychosocial problems.

**Fig. 2:**
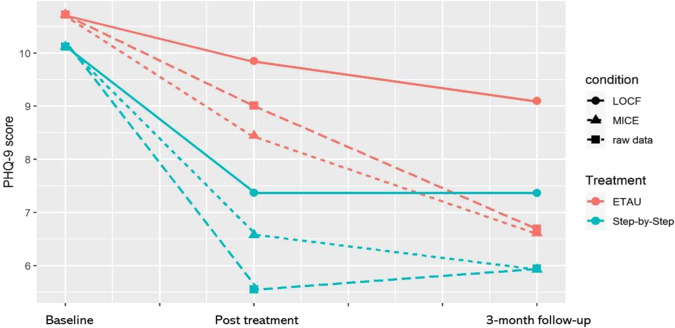
PHQ-9 scores at three time points. Changes in the primary outcome during and after the intervention in both treatment conditions.

Although the treatment gains from SbS were maintained at 3-month follow-up and there was little sign of symptom relapse for the intervention group at the follow-up (see Fig. 2), our study did not produce evidence for between-group differences in reducing depressive or anxiety symptoms at 3-month follow-up. This could be due in part to the high dropout rate at three-month follow-up, which led to reduced statistical power for the long-term effectiveness evaluation. The dropout rate reported in this study could be used as a reference in the sample size estimation of future digital interventions and our qualitative results provide insights about how future studies could reduce dropouts. Our results also indicated that regression to the mean and a floor effect might have contributed to the lower observed between groups differences at follow-up. In terms of psychological wellbeing, SbS was superior to ETAU in improving wellbeing both at posttreatment and at 3-month follow-up. This suggests that a comprehensive understanding of the long-term effectiveness of SbS could be assessed using a wider range of mental health indicators, including both symptomatic outcomes and positive outcomes, including flourishing, thriving, and resilience.

By working closely with the Student Affairs Office, the SbS trial was implemented as a free service for students in the university. This provided an opportunity to evaluate SbS as a part of the services provided to students at the university and obtain insights into wider implementation within the university setting. The overall feedback on the intervention from the qualitative evaluation was positive whereas significant limitations including mainly text-based content and long time interval between sessions that reduced treatment adherence were also mentioned. The young adult participants encountered few technical issues and were able to use the techniques with minimal guidance. They enjoyed the interactive features of the digital intervention more and can adopt the skills into daily life. However, low engagement was observed in most of the participants, along with significant dropout during the trial. According to feedback from participants, they sometimes forgot to use the app and found it difficult to maintain their engagement between sessions without regular supervision and reminders. Given the integration of artificial intelligence and other higher-end features in mobile gaming and phone use, the SbS program needs to keep up to be relevant and useful for young adults. Participants asked for more customized content that exactly fit their needs, and they expressed that regular updating of multimedia content, including videos and incorporating social network features, would make it a more attractive intervention.

Although digital interventions like SbS are effective and low-cost, poor adherence and engagement remain the biggest challenges for implementation. According to a meta-analysis of clinical trials of smartphone apps for depressive symptoms, the adjusted pooled dropout rate was 47.8% [60]. This was consistent in this trial as well, with an overall dropout of 48.4% in both groups at posttreatment. As reviewed by Christensen et al. [53], very few studies formally examined reasons for dropout from digital mental health interventions, neither did previous trials of SbS. By interviewing a sample of those who dropped out from the intervention, we found the most common reason was the lack of contact with peer supporters. There is a general trend of improved adherence within non-clinician-guided digital mental health interventions when compared with unguided interventions [61]. In our trial, student peer supporters played an important role by providing company, encouragement, support, and reminders during the intervention by making anonymous phone calls to the clients. However, a considerable number among those who dropped out reported that they failed to establish constant contact with their peer supporters. Therefore, further efforts are needed to facilitate the communication between client and the lay “coaches” for remote digital interventions. According to the interviews, social network tools could be welcomed channels for young adult users to communicate with their supporters. An alternative strategy is to integrate AI-based language models such as the ChatGPT into the digital interventions. The chatbots are now well-developed to respond to the clients any time on demand and can therefore take the role of “coach”. Also, one-fourth of those who dropped out from the intervention reported that the program contained too much content, which indicates that effort is needed to make the SbS intervention briefer while keeping its core treatment elements to improve the acceptance of the intervention among young population. Recent trial evidence demonstrated that single-session digital interventions showed good acceptance among young people [62]. In addition, our study also identified a dropout pattern that could be specific to digital interventions: most of the people who dropped out did so before the first intervention session. This means most of the participants chose to quit before they had a clear picture of the treatment content (i.e., *no shows* or *early dropouts*; [63]). This finding informs future practice to improve user engagement in digital mental health services: most efforts should be made at the recruitment stage and the very beginning of the intervention to encourage the clients to complete at least one session before they choose whether to drop out.

In addition to high dropout, the trial has some other limitations. Following the study protocol, we conducted the baseline assessments after the randomization of participants. But the dropout during baseline was much higher than we anticipated. Therefore, although we recruited adequate participants for the trial, we failed to run a formal ITT analysis and the final analytic sample was smaller than expected. The randomization should be moved after baseline assessment completion in future trials. Also, as the app was designed to only download data required for the assigned group, and the download package for the intervention group is larger than ETAU, 23 users dropped at this point and did not download the full app, which further limited the statistical power of the sample. This issue can be addressed by refining the logic of the software download or switch to other fully online delivery channels. Furthermore, the outcome assessments were reliant on self-report measures without diagnostic interviews, which is common to digital trials that emphasize the remote nature of the intervention and automated assessment. Lastly, we only followed participants until 3-months after the intervention, which limits our evaluation of the longer-term maintenance of the treatment gains and possible prevention of worsening depressive symptoms.

Despite these limitations, this was the first randomized control trial of Step-by-Step in China, and the intervention was effective at reducing depressive and anxiety symptoms among young adults. The intervention also shows a long-term effectiveness in improving psychological wellbeing. This university-based delivery model which incorporated student peer supporters and institutional stakeholders’ support (e.g., training and supervision provided by the university counseling center) could be generalized to implement digital intervention among other Chinese universities, following additional efforts to improve treatment engagement informed by this trial. This study also contributes important new knowledge for clinical research and practice by showing the potential of digital health in geographic regions with a limited mental health work force and resources.

### Supplementary information

Supplementary material

Computer codes

Interview codebook

## Data Availability

Analytical codes for quantitative analysis and code book for the qualitative are available in additional files. Clinical data are available from the corresponding author upon request.
