# Supplementary material for: A WHO digital intervention to address depression among young Chinese adults: a type 1 effectiveness-implementation randomized controlled trial
Source: Transl Psychiatry. 2024 Feb 20;14:102. doi: 10.1038/s41398-024-02812-3 (PMC10879210; doi:10.1038/s41398-024-02812-3)
Supplement: Supplementary file 1 — Supplementary material [file 41398_2024_2812_MOESM1_ESM.docx]

**Supplementary materials for article entitled:**

A WHO digital intervention to address depression among young adults in Macao, China: a type 1 effectiveness-implementation randomized controlled trial

Gen L, et al.

**Table of contents**

Methods for the sensitivity analyses 2

Supplementary Table 1. Convergence of MICE. 3

Supplementary Table 2. Results of sensitivity analyses using LOCF in Intent-to-Treat and Per protocol Samples 4

Supplementary Table 3. Results of sensitivity analyses using MICE in Intent-to-Treat and Per protocol Samples 4

Supplementary Table 4. Subgroup analyses for secondary outcomes 5

References 6

**Methods for the sensitivity analyses**

All the sensitivity analyses were conducted using R. We first used last observation carried forward (LOCF, Hamer & Simpson, 2009) to imputed missing outcome observations: missing primary outcomes at posttreatment were imputed by last observations during the weekly assessments. If weekly assessment data were also missing, then the baseline value was used. Missing primary outcomes at follow-up were imputed with the posttreatment values. If posttreatment values were missing, the values were then imputed with basline or weekly seessment data. As secondary outcomes do mot have weekly assessment, their missing value at posttreatment was imputed with their baseline values and then missing outcomes at follow-up were imputed with baseline or posttreatment data. Since the PSYCHLOPS values were 8.8% missing at baseline in the ITT sample, they were imputed with the mean value of the treatment group’s baseline PSYCHLOPS score. There was no missing data in other measures at baseline.

Multivariate imputation by chained equations (MICE) was also conducted to impute the missing values caused by dropouts, using the MICE package in R (Zhang, 2016). To impute the missing values of the outcomes, predictive mean matching (Little, 1988) was employed based on background characteristics (i.e., gender, history of past treatment, and history of mental illness) and baseline assessments of the outcomes. Convergence of imputations was checked by the value of $\hat{R}$ statistic was used as a convergence criterion, where a value less than 1.1 means convergence was achieved (Gelman & Rubin, 1992).

*Supplementary Table 1.* Convergence of MICE

|  | $\hat{\boldsymbol{R}}$**^a^ of posterior mean** | $\hat{\boldsymbol{R}}$ **of posterior variance** |
| --- | --- | --- |
| **Primary outcome** |  |  |
| PHQ_post | 0.985 | 0.999 |
| PHQ_fu | 1.014 | 1.008 |
| **Secondary outcome** |  |  |
| WHO_post | 1.005 | 1.009 |
| WHO_fu | 1.000 | 1.033 |
| GAD_post | 1.013 | 1.018 |
| GAD_fu | 1.000 | 1.006 |
| Psychlops_pre | 1.012 | 1.011 |
| Psychlops_post | 0.995 | 1.007 |
| Psychlops_fu | 1.008 | 0.996 |

^a^ The value of $\hat{R}$ statistic was used as a convergence criterion.

*Supplemnetary Table 2.* Results of sensitivity analyses using LOCF in Intent-to-Treat and Per protocol Samples

|  | Intent-to-Treat (N = 285) | | | | | | |  | Per-protocol (N = 182) | | | | | | | |
| --- | --- | --- | --- | --- | --- | --- | --- | --- | --- | --- | --- | --- | --- | --- | --- | --- |
|  | **Posttreatment** | | |  | **Follow-up** | | |  | **Posttreatment** | | |  | **Follow-up** | | |  |
|  | ***β* (95% CI)** | ***p*** | **Hedges’ *g***  **(95% CI)** |  | ***β* (95% CI)** | ***p*** | **Hedges’ *g***  **(95% CI)** |  | ***β* (95% CI)** | ***p*** | **Hedges’ *g***  **(95% CI)** |  | ***β* (95% CI)** | ***p*** | **Hedges’ *g***  **(95% CI)** |  |
| Primary outcome | |  |  |  |  |  |  |  |  |  |  |  |  |  |  |  |
| PHQ-9 | 1.52 (0.54, 2.51) | **0.003** | 0.36 (0.13, 0.60) |  | 1.16 (0.17, 2.15) | **0.022** | 0.28 (0.04, 0.51) |  | 2.66 (1.38, 3.94) | **<0.001** | 0.61 (0.37, 0.85) |  | 2.20 (0.92, 3.47) | **0.001** | 0.51 (0.27, 0.74) |  |
| Secondary outcome | |  |  |  |  |  |  |  |  |  |  |  |  |  |  |  |
| WHO-5 | -1.29 (-2.31, -0.28) | **0.013** | 0.30 (0.06, 0.53) |  | -1.36 (-2.39, -0.33) | **0.01** | 0.31 (0.08, 0.54) |  | -1.75 (-3.12, -0.39) | **0.013** | 0.38 (0.14, 0.61) |  | -2.37 (-3.74, -1.00) | **0.001** | 0.51 (0.27, 0.75) |  |
| GAD-7 | 1.09 (0.09, 2.09) | **0.033** | 0.26 (0.02, 0.49) |  | 0.79 (-0.20, 1.77) | 0.117 | 0.19 (-0.05, 0.42) |  | 1.19 (-0.15, 2.53) | 0.082 | 0.26 (0.03, 0.50) |  | 0.95 (-0.33, 2.23) | 0.149 | 0.22 (-0.02, 0.45) |  |
| PSYCHLOPS | 0.51 (-0.26, 1.29) | 0.196 | 0.16 (-0.08, 0.39) |  | 0.35 (-0.46, 1.15) | 0.397 | 0.10 (-0.13, 0.33) |  | 1.45 (0.42, 2.48) | **0.006** | 0.42 (0.18, 0.65) |  | 1.65 (0.61, 2.70) | **0.002** | 0.47 (0.23, 0.70) |  |

*Note.* GLMM was employed with adjusted covariates including time, participants, gender, history of past treatment, and history of mental illness.

*Supplemnetary Table 3.* Results of sensitivity analyses using MICE in Intent-to-Treat and Per protocol Samples

|  | Intent-to-Treat (N = 285) | | | | | | |  | Per-protocol (N = 182) | | | | | | |
| --- | --- | --- | --- | --- | --- | --- | --- | --- | --- | --- | --- | --- | --- | --- | --- |
|  | **Posttreatment** | | |  | **Follow-up** | | |  | **Posttreatment** | | |  | **Follow-up** | | |
|  | ***β* (95% CI)** | ***p*** | **Hedges’ *g***  **(95% CI)** |  | ***β* (95% CI)** | ***p*** | **Hedges’ *g***  **(95% CI)** |  | ***β* (95% CI)** | ***p*** | **Hedges’ *g***  **(95% CI)** |  | ***β* (95% CI)** | ***p*** | **Hedges’ *g***  **(95% CI)** |
| Primary outcome | |  |  |  |  |  |  |  |  |  |  |  |  |  |  |
| PHQ-9 | 1.26 (0.25, 2.28) | **0.016** | 0.41 (0.18, 0.65) |  | 0.54 (-0.40, 1.48) | 0.26 | 0.18 (-0.06, 0.41) |  | 2.14 (0.89, 3.38) | **0.001** | 0.52 (0.28, 0.76) |  | 1.09 (-0.15, 2.32) | 0.086 | 0.27 (0.04, 0.50) |
| Secondary outcome | |  |  |  |  |  |  |  |  |  |  |  |  |  |  |
| WHO-5 | -1.13 (-2.25, -0.01) | 0.051 | 0.37 (0.14, 0.61) |  | -1.16 (-2.24, -0.09) | **0.036** | 0.37 (0.13, 0.60) |  | -1.56 (-2.91, -0.21) | **0.025** | 0.35 (-0.12, 0.58) |  | -1.83 (-3.17, -0.48) | **0.009** | 0.41 (-0.17, 0.64) |
| GAD-7 | 0.99 (0.03, 1.94) | **0.043** | 0.20 (-0.03, 0.44) |  | 0.4 (-0.47, 1.27) | 0.369 | 0.09 (-0.15, 0.32) |  | 1.02 (-0.32, 2.37) | 0.138 | 0.23 (-0.01, 0.46) |  | 0.28 (-0.96, 1.53) | 0.658 | 0.07 (-0.17, 0.30) |
| PSYCHLOPS | 0.61 (-0.18, 1.40) | 0.134 | 0.19 (-0.04, 0.43) |  | 0.57 (-0.28, 1.42) | 0.19 | 0.27 (0.03, 0.50) |  | 0.96 (-0.11, 2.04) | 0.08 | 0.28 (0.04, 0.51) |  | 1.09 (0.03, 2.15) | **0.045** | 0.33 (0.09, 0.56) |

*Note.* GLMM was employed with adjusted covariates including time, participants, gender, history of past treatment, and history of mental illness.

|  |  | **Subgroups** | **ITT (n = 285)** | | |
| --- | --- | --- | --- | --- | --- |
|  |  |  | ***β* (95% CI)** | ***p*** | **Hedges’ *g***  **(95% CI)** |
| **Posttreatment** |  |  |  |  |  |
|  | **WHO-5** | **Gender ^a^** |  |  |  |
|  |  | Female (n = 197) | -1.01 (-2.23, 0.21) | 0.105 | 0.23 (-0.04, 0.50) |
|  |  | Male (n = 88) | -1.99 (-3.85, -0.13) | **0.039** | 0.46 (0.05, 0.86) |
|  | **GAD-7** | **Gender** | |  |  |
|  |  | Female (n = 197) | 0.85 (-0.41, 2.09) | 0.187 | 0.19 (-0.09, 0.46) |
|  |  | Male (n = 88) | 1.62 (-0.02, 3.26) | 0.056 | 0.44 (0.04, 0.85) |
|  | **PSYCHLOPS** | **Gender** |  |  |  |
|  |  | Female (n = 197) | 0.60 (-0.42, 1.62) | 0.251 | 0.10 (-0.10, 0.44) |
|  |  | Male (n = 88) | 0.88 (-0.46, 2.22) | 0.203 | 0.30 (-0.10, 0.70) |
| **Follow-up** |  |  |  |  |  |
|  | **WHO-5** | **Gender** |  |  |  |
|  |  | Female (n = 197) | -1.00 (-2.20, 0.20) | 0.103 | 0.23 (-0.04, 0.50) |
|  |  | Male (n = 88) | -2.02 (-3.97, -0.06) | **0.046** | 0.44 (0.03, -0.84) |
|  | **GAD-7** | **Gender** | |  |  |
|  |  | Female (n = 197) | 0.38 (-0.77, 1.53) | 0.517 | 0.09 (-0.18, 0.36) |
|  |  | Male (n = 88) | 1.08 (-0.75, 2.91) | 0.251 | 0.25 (-0.15, 0.65) |
|  | **PSYCHLOPS** | **Gender** |  |  |  |
|  |  | Female (n = 197) | 0.60 (-0.42, 1.61) | 0.252 | 0.18 (-0.10, 0.45) |
|  |  | Male (n = 88) | 0.52 (-0.95, 1.99) | 0.491 | 0.16 (-0.25, 0.56) |

*Supplemnetary Table 4.* Subgroup analyses of the effectiveness of Step by Step in treating depression symptoms in the Intent-to-Treat Sample

*Note.* ^a^ Adjusted covariates included time, participants, history of past treatment, and history of mental illness.

**Reference**

Gelman, A., & Rubin, D. B. (1992). Inference from Iterative Simulation Using Multiple Sequences. *Statistical Science*, *7*(4), 457–472.

Hamer, R. M., & Simpson, P. M. (2009). Last observation carried forward versus mixed models in the analysis of psychiatric clinical trials. *The American Journal of Psychiatry*, *166*(6), 639–641.

Little, R. J. A. (1988). Missing-Data Adjustments in Large Surveys. *Journal of Business & Economic Statistics*, *6*(3), 287.

Zhang, Z. (2016). Multiple imputation with multivariate imputation by chained equation (MICE) package. *Annals of Translational Medicine*, *4*(2).
