# Supplementary material for: A WHO digital intervention to address depression among young Chinese adults: a type 1 effectiveness-implementation randomized controlled trial
Source: Transl Psychiatry. 2024 Feb 20;14:102. doi: 10.1038/s41398-024-02812-3 (PMC10879210; doi:10.1038/s41398-024-02812-3)
Supplement: Supplementary file 2 — Computer codes [file 41398_2024_2812_MOESM2_ESM.docx]

###缺失数据###

options(scipen = 200)

library(tidyverse)

# library(mi) #不好用

library(mice)

library(ggplot2)

library(misty)

library(tableone)

library(miceadds)#提供Rhat的函数

library(lme4)#glmm

library(lmerTest)# lmmtest

library(broom.mixed) ##mixed model pool

library(Hmisc)##impute函数

library(gridExtra)

##数据清理

sbs_dat <- read.csv("SbS_Macao_effectiveness0914.csv")

# colnames(sbs_dat)[4:9] <- c("Gender", "Age", "Nationality","Education status", "Marriage","Born in Macao")

# colnames(sbs_dat)[14] <- "history of past treatment"

# colnames(sbs_dat)[12] <- "history of mental health"

##miss pattern

# miss_pattern<- md.pattern(sbs_dat) #md.pattern

# x <- print(miss_pattern)

##不转换列的顺序

myfun <-function (x, plot = TRUE, rotate.names = FALSE)

{

if (!(is.matrix(x) || is.data.frame(x))) {

stop("Data should be a matrix or dataframe")

}

if (ncol(x) < 2) {

stop("Data should have at least two columns")

}

R <- is.na(x)

nmis <- colSums(R)

# R <- matrix(R[, order(nmis)], dim(x))

pat <- apply(R, 1, function(x) paste(as.numeric(x), collapse = ""))

sortR <- matrix(R[order(pat), ], dim(x))

if (nrow(x) == 1) {

mpat <- is.na(x)

}

else {

mpat <- sortR[!duplicated(sortR), ]

}

if (all(!is.na(x))) {

cat(" /\\ /\\\n{ `---' }\n{ O O }\n==> V <==")

cat(" No need for mice. This data set is completely observed.\n")

cat(" \\ \\|/ /\n `-----'\n\n")

mpat <- t(as.matrix(mpat, byrow = TRUE))

rownames(mpat) <- table(pat)

}

else {

if (is.null(dim(mpat))) {

mpat <- t(as.matrix(mpat))

}

rownames(mpat) <- table(pat)

}

r <- cbind(abs(mpat - 1), rowSums(mpat))

r <- rbind(r, c(nmis[order(nmis)], sum(nmis)))

if (plot) {

op <- par(mar = c(1, 1, 1, 1) + 0.1)

on.exit(par(op))

plot.new()

if (is.null(dim(sortR[!duplicated(sortR), ]))) {

R <- t(as.matrix(r[1:nrow(r) - 1, 1:ncol(r) - 1]))

}

else {

if (is.null(dim(R))) {

R <- t(as.matrix(R))

}

R <- r[1:nrow(r) - 1, 1:ncol(r) - 1]

}

if (rotate.names) {

adj <- c(0, 0.5)

srt <- 90

length_of_longest_colname <- max(nchar(colnames(r)))/2.6

plot.window(xlim = c(-1, ncol(R) + 1), ylim = c(-1,

nrow(R) + length_of_longest_colname), asp = 1)

}

else {

adj <- c(0.5, 0)

srt <- 0

plot.window(xlim = c(-1, ncol(R) + 1), ylim = c(-1,

nrow(R) + 1), asp = 1)

}

M <- cbind(c(row(R)), c(col(R))) - 1

shade <- ifelse(R[nrow(R):1, ], mdc(1), mdc(2))

rect(M[, 2], M[, 1], M[, 2] + 1, M[, 1] + 1, col = shade)

for (i in 1:ncol(R)) {

text(i - 0.5, nrow(R) + 0.3, colnames(r)[i], adj = adj,

srt = srt,cex = 0.5)

text(i - 0.5, -0.3, nmis[][i])#order(nmis)

}

for (i in 1:nrow(R)) {

text(ncol(R) + 0.3, i - 0.5, r[(nrow(r) - 1):1, ncol(r)][i],

adj = 0)

text(-0.3, i - 0.5, rownames(r)[(nrow(r) - 1):1][i],

adj = 1)

}

text(ncol(R) + 0.3, -0.3, r[nrow(r), ncol(r)])

return(r)

}

else {

return(r)

}

}

miss_pattern<- myfun(sbs_dat)

write.csv(miss_pattern,file = "missing.csv")

###检测是否为MNAR

sbs_dat2 <- read.csv("SbS_Macao_effectiveness -complete.csv")

na.test(sbs_dat2[,c(-1,-20,-ncol(sbs_dat2))])##20为PHQ imputation

colnames(sbs_dat2)

# ###奇异矩阵?

# ?na.test

##观察数据缺失者的处理分布

missing_colname <- colnames(sbs_dat)[colSums(is.na(sbs_dat))>0]##提出缺失列

miss_by_treat <- NULL

for (i in 1:length(missing_colname)) {

contingency_table <- table(sbs_dat[,"Treatment"],

is.na(sbs_dat[,missing_colname[i]]))

test <- fisher.test(contingency_table)

test$p.value

miss_by_treat <- rbind(miss_by_treat,cbind(contingency_table,c(test$p.value,NA), c(missing_colname[i],NA)))

}

#导出结果

write.csv(miss_by_treat,file = "miss_by_treat.csv")

##比较PHQ_pre的分布

sbs_dat2 <-sbs_dat

sbs_dat2$index <-is.na(sbs_dat[,14])

x<- sbs_dat[is.na(sbs_dat[,14]),]$PHQ_pre

y<- sbs_dat[!is.na(sbs_dat[,14]),]$PHQ_pre

x_lim <- 0:30

ggplot(sbs_dat2,aes(x=PHQ_pre))+

geom_density(aes(color = index))+xlim(0,30)

#1. table1 ------------------------------------------------------------------

#合并分类####

#education Finish PG及以上合并

sbs_dat <- sbs_dat%>%mutate(.,education2= case_when(Demog4==2~"Finish high school",

Demog4==4~"UG student",

is.na(Demog4) ~ "NA",

T ~ "Finish UG or above"))

sbs_dat$education2 <- with(sbs_dat,ifelse(education2=="NA",NA,education2))

#marriage

sbs_dat <- sbs_dat%>%mutate(.,marriage2= case_when(Demog5==1~"Single",

is.na(Demog5) ~ "NA",

T ~ "Non-single"))

sbs_dat$marriage2 <- with(sbs_dat,ifelse(marriage2=="NA",NA,marriage2))

# table(sbs_dat$marriage2,useNA = "always")

##itt

colnames(sbs_dat)

sbs_dat[,c(1:4,6:17,31,32)] <- apply(sbs_dat[,c(1:4,6:17,31,32)],2,as.factor)

str(sbs_dat)

head(sbs_dat)

table1<- CreateTableOne(colnames(sbs_dat)[-1],strata = "Treatment",data = sbs_dat,addOverall = T,includeNA = T)

myt1 <- print(table1,showAllLevels = T,missing = T)

##remove Baseline uncomplete

sbs_dat_basecom <- sbs_dat%>%filter(.,BaselineCompletion==1)

table_basecom<- CreateTableOne(colnames(sbs_dat_basecom)[-1],strata = "Treatment",data = sbs_dat_basecom,addOverall = T,includeNA = T)

myt1_basecom <- print(table_basecom,showAllLevels = T,missing = T)

write.csv(myt1_basecom,file = "table1_baselinecompletion.csv")

# 2.multiple imputation ----------------------------------------------------

ana_dat <- sbs_dat_basecom

ana_dat_impu <- ana_dat%>%select(ID,Treatment,Demog1,Demog9,Demog11,

PHQ_pre,PHQ_post,PHQ_fu,

WHO_pre,WHO_post,WHO_fu,

GAD_pre,GAD_post,GAD_fu,

Psychlops_pre,Psychlops_post,Psychlops_fu,

Completion)

# colnames(ana_dat)

impu_var <- c("PHQ_pre","PHQ_post","PHQ_fu",

"WHO_pre","WHO_post","WHO_fu",

"GAD_pre","GAD_post","GAD_fu",

"Psychlops_pre","Psychlops_post","Psychlops_fu")

imp <- mice(ana_dat_impu,seed = 1234)

pred<- imp$predictorMatrix

# quick_pred<- quickpred(ana_dat_impu) #quick predictor 不好用

pred[setdiff(rownames(pred),impu_var),] <-0#不必填补的

pred[,impu_var[-c(1,4,7,10)]] <- 0 #predictor不需要post和fu

pred[,"Treatment"] <- 0

pred[,"Completion"] <- 0##

pred#

imp <- mice(ana_dat_impu, predictorMatrix = pred, m=10,maxit = 20,seed = 1234,

printFlag = F,method = "pmm")

imp$method

###计算Rhat

Rhat.mice(imp)

write.csv(Rhat.mice(imp),"Rhat.mice.csv")

##chain plot

plot(imp,c("PHQ_post","PHQ_fu"))

dev.new()

plot(imp,c("WHO_post","WHO_fu"))

dev.new()

plot(imp,c("GAD_post","GAD_fu")) #GAD_post还比较收敛

dev.new()

plot(imp,c("Psychlops_pre","Psychlops_post","Psychlops_fu"))

# 3. modeling -------------------------------------------------------------

#处理post_impu数据格式####

mid_impu_data <- imp$data

##写循环存结果

#3.1预后效应####

GLMM_pro_fun <- function(data,subset_index=T){#subset_index为logic vector，指代用哪个子集

##将impute数据存入原数据集中

glmer_fit_PHQ <- list()

glmer_fit_WHO <- list()

glmer_fit_GAD <- list()

glmer_fit_Psychlops <- list()

#跑循环####

m=10

for (j in 1:m) { #10为m

for (i in 1:length(impu_var)) {

data[,impu_var[i]] <- impute(mid_impu_data[,impu_var[i]],

imp$imp[[impu_var[i]]][,j])[subset_index]

}

####获得gather data: pre & post

data_gather <- gather(data,key=Time, value= PHQ, "PHQ_pre","PHQ_post")

data_gather[,"WHO"] <- c(data[,"WHO_pre"], data[,"WHO_post"])

data_gather[,"GAD"] <- c(data[,"GAD_pre"], data[,"GAD_post"])

data_gather[,"Psychlops"] <- c(data[,"Psychlops_pre"], data[,"Psychlops_post"])

#结局转化为2分类 PHQ GAD

data_gather <- data_gather%>%

mutate(., PHQ2 = ifelse(PHQ>=10,1,0))%>%

mutate(., GAD2 = ifelse(GAD>=10,1,0))

##modeling###

glmer_fit_PHQ[[j]] <- lmer(PHQ ~ Treatment+Time+Demog1+Demog9+Demog11+(1|ID),

data =data_gather)

glmer_fit_WHO[[j]] <- lmer(WHO ~ Treatment+Time+Demog1+Demog9+Demog11+(1|ID),

data =data_gather)

glmer_fit_GAD[[j]] <- lmer(GAD ~ Treatment+Time+Demog1+Demog9+Demog11+(1|ID),

data =data_gather)

glmer_fit_Psychlops[[j]] <-lmer(Psychlops ~ Treatment+Time+Demog1+Demog9+Demog11+(1|ID),

data =data_gather)

}

summary(pool(glmer_fit_PHQ))

summary(pool(glmer_fit_WHO))

summary(pool(glmer_fit_GAD))

summary(pool(glmer_fit_Psychlops))

res_mi_pro <- list(summary(pool(glmer_fit_PHQ)),

summary(pool(glmer_fit_WHO)),

summary(pool(glmer_fit_GAD)),

summary(pool(glmer_fit_Psychlops)))

print(res_mi_pro)

}

res_mi_pro <- GLMM_pro_fun(mid_impu_data)

#3.2长期效应####

GLMM_pro_fun_long <- function(data,subset_index=T){

##将impute数据存入原数据集中

glmer_fit_PHQ_long <- list()

glmer_fit_WHO_long <- list()

glmer_fit_GAD_long <- list()

glmer_fit_Psychlops_long <- list()

#跑循环####

m=10

for (j in 1:m) { #10为m

for (i in 1:length(impu_var)) {

data[,impu_var[i]] <- impute(mid_impu_data[,impu_var[i]],

imp$imp[[impu_var[i]]][,j])[subset_index]

}

####获得gather data: pre & fu

data_gather <- gather(data,key=Time, value= PHQ, "PHQ_pre","PHQ_fu")

data_gather[,"WHO"] <- c(data[,"WHO_pre"], data[,"WHO_fu"])

data_gather[,"GAD"] <- c(data[,"GAD_pre"], data[,"GAD_fu"])

data_gather[,"Psychlops"] <- c(data[,"Psychlops_pre"], data[,"Psychlops_fu"])

#结局转化为2分类 PHQ GAD

data_gather <- data_gather%>%

mutate(., PHQ2 = ifelse(PHQ>=10,1,0))%>%

mutate(., GAD2 = ifelse(GAD>=10,1,0))

##modeling###

glmer_fit_PHQ_long[[j]] <- lmer(PHQ ~ Treatment+Time+Demog1+Demog9+Demog11+(1|ID),

data =data_gather)

glmer_fit_WHO_long[[j]] <- lmer(WHO ~ Treatment+Time+Demog1+Demog9+Demog11+(1|ID),

data =data_gather)

glmer_fit_GAD_long[[j]] <- lmer(GAD ~ Treatment+Time+Demog1+Demog9+Demog11+(1|ID),

data =data_gather)

glmer_fit_Psychlops_long[[j]] <-lmer(Psychlops ~ Treatment+Time+Demog1+Demog9+Demog11+(1|ID),

data =data_gather)

}

res_mi_long <- list(summary(pool(glmer_fit_PHQ_long)),

summary(pool(glmer_fit_WHO_long)),

summary(pool(glmer_fit_GAD_long)),

summary(pool(glmer_fit_Psychlops_long)))

print(res_mi_long)

}

res_mi_long <- GLMM_pro_fun_long(mid_impu_data)

##3.3 effect size####

#编写 cohen's d hedgesd of a regression函数

#n1 n2

n1=132;n2=153

#计算cohen's d

cohens_d_regression <- function(t,n1,n2,df){# Effect size, confidence interval and statistical significance: a practical guide for biologists

#计算cohen's d

d <- t*(n1+n2)/(sqrt(n1*n2)*sqrt(df)) #公式10

#计算Hedges d

d_hedges <- d *(1-(3/(4*(n1+n2-2)-1))) #公式14

#计算se for cohen's d 公式16

index1 <- (n1+n2-1)/ (n1+n2-3)

index2 <- (4/(n1+n2))*(1+d^2/8)

se <- sqrt(index1*index2)

#计算se for Hedges d 公式17

index1 <- (n1+n2)/(n1*n2)

index2 <- d_hedges^2/(2*(n1+n2-2))

se_hedges <- sqrt(index1+index2)

res <- c(d,se,d-1.96*se,d+1.96*se,d_hedges, se_hedges, d_hedges-1.96*se_hedges, d_hedges+1.96*se_hedges)

names(res) <- c("cohen's d", "se", "95%CI-lower", "95%CI-upper","hedges' d", "se_ hedges", "l", "u")

print(res)

}

COHENS_D <- function(data){

COHENS_D_mi <-data.frame()

for (i in 1:4) {

COHENS_D_mi <-rbind(COHENS_D_mi, with(data[[i]][2,], cohens_d_regression(t=statistic,n1=n1,n2=n2,df=df))

)

}

COHENS_D_mi <- round(COHENS_D_mi,digits = 3)

colnames(COHENS_D_mi) <- c("cohensd", "se_cohens", "lci_cohens", "uci_cohens",

"hedgesd","se_hedges", "lci_hedges","uci_hedges")

COHENS_D_mi$effectsize_cohens <- with(COHENS_D_mi,paste(cohensd, " (",lci_cohens,", ",uci_cohens,")",sep = ""))

COHENS_D_mi$effectsize_hedges <- with(COHENS_D_mi,paste(hedgesd, " (",lci_hedges,", ",uci_hedges,")",sep = ""))

COHENS_D_mi$indicator <- c("PHQ","WHO","GAD", "Psychlops")

print(COHENS_D_mi)

}

#预后

# COHENS_D_mi_pro <- COHENS_D(res_mi_pro)

#长期

# COHENS_D_mi_long <- COHENS_D(res_mi_long)

# 4.1 subgroup analysis for post ---------------------------------------------------------------------

##4.1.1 gender####

#female####

mid_impu_data_female <- mid_impu_data%>%filter(.,Demog1==2)

data <- mid_impu_data_female

subset_index <- with(mid_impu_data,Demog1==2)

##将impute数据存入原数据集中

glmer_fit_PHQ <- list()

glmer_fit_WHO <- list()

glmer_fit_GAD <- list()

glmer_fit_Psychlops <- list()

#跑循环####

m=10

for (j in 1:m) { #10为m

for (i in 1:length(impu_var)) {

data[,impu_var[i]] <- impute(mid_impu_data[,impu_var[i]],

imp$imp[[impu_var[i]]][,j])[subset_index]

}

####获得gather data: pre & post

data_gather <- gather(data,key=Time, value= PHQ, "PHQ_pre","PHQ_post")

data_gather[,"WHO"] <- c(data[,"WHO_pre"], data[,"WHO_post"])

data_gather[,"GAD"] <- c(data[,"GAD_pre"], data[,"GAD_post"])

data_gather[,"Psychlops"] <- c(data[,"Psychlops_pre"], data[,"Psychlops_post"])

#结局转化为2分类 PHQ GAD

data_gather <- data_gather%>%

mutate(., PHQ2 = ifelse(PHQ>=10,1,0))%>%

mutate(., GAD2 = ifelse(GAD>=10,1,0))

##modeling###

#少9

glmer_fit_PHQ[[j]] <- lmer(PHQ ~ Treatment+Time+Demog11+(1|ID),

data =data_gather)

glmer_fit_WHO[[j]] <- lmer(WHO ~ Treatment+Time+Demog11+(1|ID),

data =data_gather)

glmer_fit_GAD[[j]] <- lmer(GAD ~ Treatment+Time+Demog11+(1|ID),

data =data_gather)

glmer_fit_Psychlops[[j]] <-lmer(Psychlops ~ Treatment+Time+Demog11+(1|ID),

data =data_gather)

}

res_mi_pro_female <- list(summary(pool(glmer_fit_PHQ)),

summary(pool(glmer_fit_WHO)),

summary(pool(glmer_fit_GAD)),

summary(pool(glmer_fit_Psychlops)))

print(res_mi_pro_female)

#male####

mid_impu_data_male <- mid_impu_data%>%filter(.,Demog1==1)

data <- mid_impu_data_male

subset_index <- with(mid_impu_data,Demog1==1)

##将impute数据存入原数据集中

glmer_fit_PHQ <- list()

glmer_fit_WHO <- list()

glmer_fit_GAD <- list()

glmer_fit_Psychlops <- list()

#跑循环####

m=10

for (j in 1:m) { #10为m

for (i in 1:length(impu_var)) {

data[,impu_var[i]] <- impute(mid_impu_data[,impu_var[i]],

imp$imp[[impu_var[i]]][,j])[subset_index]

}

####获得gather data: pre & post

data_gather <- gather(data,key=Time, value= PHQ, "PHQ_pre","PHQ_post")

data_gather[,"WHO"] <- c(data[,"WHO_pre"], data[,"WHO_post"])

data_gather[,"GAD"] <- c(data[,"GAD_pre"], data[,"GAD_post"])

data_gather[,"Psychlops"] <- c(data[,"Psychlops_pre"], data[,"Psychlops_post"])

#结局转化为2分类 PHQ GAD

data_gather <- data_gather%>%

mutate(., PHQ2 = ifelse(PHQ>=10,1,0))%>%

mutate(., GAD2 = ifelse(GAD>=10,1,0))

##modeling###

#少9

glmer_fit_PHQ[[j]] <- lmer(PHQ ~ Treatment+Time+Demog9+Demog11+(1|ID),

data =data_gather)

glmer_fit_WHO[[j]] <- lmer(WHO ~ Treatment+Time+Demog9+Demog11+(1|ID),

data =data_gather)

glmer_fit_GAD[[j]] <- lmer(GAD ~ Treatment+Time+Demog9+Demog11+(1|ID),

data =data_gather)

glmer_fit_Psychlops[[j]] <-lmer(Psychlops ~ Treatment+Demog9+Time+Demog11+(1|ID),

data =data_gather)

}

res_mi_pro_male <- list(summary(pool(glmer_fit_PHQ)),

summary(pool(glmer_fit_WHO)),

summary(pool(glmer_fit_GAD)),

summary(pool(glmer_fit_Psychlops)))

print(res_mi_pro_male)

##4.1.2 depression severity####

#mild####

mid_impu_data_mild <- mid_impu_data%>%filter(.,PHQ_pre>=5&PHQ_pre<10)

data <- mid_impu_data_mild

subset_index <- with(mid_impu_data,PHQ_pre>=5&PHQ_pre<10)

##将impute数据存入原数据集中

glmer_fit_PHQ <- list()

glmer_fit_WHO <- list()

glmer_fit_GAD <- list()

glmer_fit_Psychlops <- list()

#跑循环####

m=10

for (j in 1:m) { #10为m

for (i in 1:length(impu_var)) {

data[,impu_var[i]] <- impute(mid_impu_data[,impu_var[i]],

imp$imp[[impu_var[i]]][,j])[subset_index]

}

####获得gather data: pre & post

data_gather <- gather(data,key=Time, value= PHQ, "PHQ_pre","PHQ_post")

data_gather[,"WHO"] <- c(data[,"WHO_pre"], data[,"WHO_post"])

data_gather[,"GAD"] <- c(data[,"GAD_pre"], data[,"GAD_post"])

data_gather[,"Psychlops"] <- c(data[,"Psychlops_pre"], data[,"Psychlops_post"])

#结局转化为2分类 PHQ GAD

data_gather <- data_gather%>%

mutate(., PHQ2 = ifelse(PHQ>=10,1,0))%>%

mutate(., GAD2 = ifelse(GAD>=10,1,0))

##modeling###

#少9

glmer_fit_PHQ[[j]] <- lmer(PHQ ~ Treatment+Time+Demog1+Demog11+(1|ID),

data =data_gather)

glmer_fit_WHO[[j]] <- lmer(WHO ~ Treatment+Time+Demog1+Demog11+(1|ID),

data =data_gather)

glmer_fit_GAD[[j]] <- lmer(GAD ~ Treatment+Time+Demog1+Demog11+(1|ID),

data =data_gather)

glmer_fit_Psychlops[[j]] <-lmer(Psychlops ~ Treatment+Time+Demog1+Demog11+(1|ID),

data =data_gather)

}

res_mi_pro_mild <- list(summary(pool(glmer_fit_PHQ)),

summary(pool(glmer_fit_WHO)),

summary(pool(glmer_fit_GAD)),

summary(pool(glmer_fit_Psychlops)))

print(res_mi_pro_mild)

#moderate####

mid_impu_data_moderate <- mid_impu_data%>%filter(.,PHQ_pre>=10&PHQ_pre<15)

data <- mid_impu_data_moderate

subset_index <- with(mid_impu_data,PHQ_pre>=10&PHQ_pre<15)

##将impute数据存入原数据集中

glmer_fit_PHQ <- list()

glmer_fit_WHO <- list()

glmer_fit_GAD <- list()

glmer_fit_Psychlops <- list()

#跑循环####

m=10

for (j in 1:m) { #10为m

for (i in 1:length(impu_var)) {

data[,impu_var[i]] <- impute(mid_impu_data[,impu_var[i]],

imp$imp[[impu_var[i]]][,j])[subset_index]

}

####获得gather data: pre & post

data_gather <- gather(data,key=Time, value= PHQ, "PHQ_pre","PHQ_post")

data_gather[,"WHO"] <- c(data[,"WHO_pre"], data[,"WHO_post"])

data_gather[,"GAD"] <- c(data[,"GAD_pre"], data[,"GAD_post"])

data_gather[,"Psychlops"] <- c(data[,"Psychlops_pre"], data[,"Psychlops_post"])

#结局转化为2分类 PHQ GAD

data_gather <- data_gather%>%

mutate(., PHQ2 = ifelse(PHQ>=10,1,0))%>%

mutate(., GAD2 = ifelse(GAD>=10,1,0))

##modeling###

#少9和11

glmer_fit_PHQ[[j]] <- lmer(PHQ ~ Treatment+Time+Demog1+(1|ID),

data =data_gather)

glmer_fit_WHO[[j]] <- lmer(WHO ~ Treatment+Time+Demog1+(1|ID),

data =data_gather)

glmer_fit_GAD[[j]] <- lmer(GAD ~ Treatment+Time+Demog1+(1|ID),

data =data_gather)

glmer_fit_Psychlops[[j]] <-lmer(Psychlops ~ Treatment+Time+Demog1+(1|ID),

data =data_gather)

}

res_mi_pro_moderate <- list(summary(pool(glmer_fit_PHQ)),

summary(pool(glmer_fit_WHO)),

summary(pool(glmer_fit_GAD)),

summary(pool(glmer_fit_Psychlops)))

print(res_mi_pro_moderate)

#high####

mid_impu_data_high <- mid_impu_data%>%filter(.,PHQ_pre>=15)

data <- mid_impu_data_high

subset_index <- with(mid_impu_data,PHQ_pre>=15)

##将impute数据存入原数据集中

glmer_fit_PHQ <- list()

glmer_fit_WHO <- list()

glmer_fit_GAD <- list()

glmer_fit_Psychlops <- list()

#跑循环####

m=10

for (j in 1:m) { #10为m

for (i in 1:length(impu_var)) {

data[,impu_var[i]] <- impute(mid_impu_data[,impu_var[i]],

imp$imp[[impu_var[i]]][,j])[subset_index]

}

####获得gather data: pre & post

data_gather <- gather(data,key=Time, value= PHQ, "PHQ_pre","PHQ_post")

data_gather[,"WHO"] <- c(data[,"WHO_pre"], data[,"WHO_post"])

data_gather[,"GAD"] <- c(data[,"GAD_pre"], data[,"GAD_post"])

data_gather[,"Psychlops"] <- c(data[,"Psychlops_pre"], data[,"Psychlops_post"])

#结局转化为2分类 PHQ GAD

data_gather <- data_gather%>%

mutate(., PHQ2 = ifelse(PHQ>=10,1,0))%>%

mutate(., GAD2 = ifelse(GAD>=10,1,0))

##modeling###

glmer_fit_PHQ[[j]] <- lmer(PHQ ~ Treatment+Time+Demog1+Demog9+Demog11+(1|ID),

data =data_gather)

glmer_fit_WHO[[j]] <-lmer(WHO ~ Treatment+Time+Demog1+Demog9+Demog11+(1|ID),

data =data_gather)

glmer_fit_GAD[[j]] <-lmer(GAD ~ Treatment+Time+Demog1+Demog9+Demog11+(1|ID),

data =data_gather)

glmer_fit_Psychlops[[j]] <-lmer(Psychlops ~ Treatment+Time+Demog1+Demog9+Demog11+(1|ID),

data =data_gather)

}

res_mi_pro_high <- list(summary(pool(glmer_fit_PHQ)),

summary(pool(glmer_fit_WHO)),

summary(pool(glmer_fit_GAD)),

summary(pool(glmer_fit_Psychlops)))

print(res_mi_pro_high)

# 4.2 subgroup analysis for fu ---------------------------------------------------------------------

##4.2.1 gender####

#female####

mid_impu_data_female <- mid_impu_data%>%filter(.,Demog1==2)

data <- mid_impu_data_female

subset_index <- with(mid_impu_data,Demog1==2)

##将impute数据存入原数据集中

glmer_fit_PHQ <- list()

glmer_fit_WHO <- list()

glmer_fit_GAD <- list()

glmer_fit_Psychlops <- list()

#跑循环####

m=10

for (j in 1:m) { #10为m

for (i in 1:length(impu_var)) {

data[,impu_var[i]] <- impute(mid_impu_data[,impu_var[i]],

imp$imp[[impu_var[i]]][,j])[subset_index]

}

####获得gather data: pre & fu

data_gather <- gather(data,key=Time, value= PHQ, "PHQ_pre","PHQ_fu")

data_gather[,"WHO"] <- c(data[,"WHO_pre"], data[,"WHO_fu"])

data_gather[,"GAD"] <- c(data[,"GAD_pre"], data[,"GAD_fu"])

data_gather[,"Psychlops"] <- c(data[,"Psychlops_pre"], data[,"Psychlops_fu"])

#结局转化为2分类 PHQ GAD

data_gather <- data_gather%>%

mutate(., PHQ2 = ifelse(PHQ>=10,1,0))%>%

mutate(., GAD2 = ifelse(GAD>=10,1,0))

##modeling###

#少9

glmer_fit_PHQ[[j]] <- lmer(PHQ ~ Treatment+Time+Demog11+(1|ID),

data =data_gather)

glmer_fit_WHO[[j]] <- lmer(WHO ~ Treatment+Time+Demog11+(1|ID),

data =data_gather)

glmer_fit_GAD[[j]] <- lmer(GAD ~ Treatment+Time+Demog11+(1|ID),

data =data_gather)

glmer_fit_Psychlops[[j]] <-lmer(Psychlops ~ Treatment+Time+Demog11+(1|ID),

data =data_gather)

}

res_mi_long_female <- list(summary(pool(glmer_fit_PHQ)),

summary(pool(glmer_fit_WHO)),

summary(pool(glmer_fit_GAD)),

summary(pool(glmer_fit_Psychlops)))

print(res_mi_long_female)

#male####

mid_impu_data_male <- mid_impu_data%>%filter(.,Demog1==1)

data <- mid_impu_data_male

subset_index <- with(mid_impu_data,Demog1==1)

##将impute数据存入原数据集中

glmer_fit_PHQ <- list()

glmer_fit_WHO <- list()

glmer_fit_GAD <- list()

glmer_fit_Psychlops <- list()

#跑循环####

m=10

for (j in 1:m) { #10为m

for (i in 1:length(impu_var)) {

data[,impu_var[i]] <- impute(mid_impu_data[,impu_var[i]],

imp$imp[[impu_var[i]]][,j])[subset_index]

}

####获得gather data: pre & fu

data_gather <- gather(data,key=Time, value= PHQ, "PHQ_pre","PHQ_fu")

data_gather[,"WHO"] <- c(data[,"WHO_pre"], data[,"WHO_fu"])

data_gather[,"GAD"] <- c(data[,"GAD_pre"], data[,"GAD_fu"])

data_gather[,"Psychlops"] <- c(data[,"Psychlops_pre"], data[,"Psychlops_fu"])

#结局转化为2分类 PHQ GAD

data_gather <- data_gather%>%

mutate(., PHQ2 = ifelse(PHQ>=10,1,0))%>%

mutate(., GAD2 = ifelse(GAD>=10,1,0))

##modeling###

#少9

glmer_fit_PHQ[[j]] <- lmer(PHQ ~ Treatment+Time+Demog9+Demog11+(1|ID),

data =data_gather)

glmer_fit_WHO[[j]] <- lmer(WHO ~ Treatment+Time+Demog9+Demog11+(1|ID),

data =data_gather)

glmer_fit_GAD[[j]] <- lmer(GAD ~ Treatment+Time+Demog9+Demog11+(1|ID),

data =data_gather)

glmer_fit_Psychlops[[j]] <-lmer(Psychlops ~ Treatment+Demog9+Time+Demog11+(1|ID),

data =data_gather)

}

res_mi_long_male <- list(summary(pool(glmer_fit_PHQ)),

summary(pool(glmer_fit_WHO)),

summary(pool(glmer_fit_GAD)),

summary(pool(glmer_fit_Psychlops)))

print(res_mi_long_male)

##4.2.2 depression severity####

#mild####

mid_impu_data_mild <- mid_impu_data%>%filter(.,PHQ_pre>=5&PHQ_pre<10)

data <- mid_impu_data_mild

subset_index <- with(mid_impu_data,PHQ_pre>=5&PHQ_pre<10)

##将impute数据存入原数据集中

glmer_fit_PHQ <- list()

glmer_fit_WHO <- list()

glmer_fit_GAD <- list()

glmer_fit_Psychlops <- list()

#跑循环####

m=10

for (j in 1:m) { #10为m

for (i in 1:length(impu_var)) {

data[,impu_var[i]] <- impute(mid_impu_data[,impu_var[i]],

imp$imp[[impu_var[i]]][,j])[subset_index]

}

####获得gather data: pre & fu

data_gather <- gather(data,key=Time, value= PHQ, "PHQ_pre","PHQ_fu")

data_gather[,"WHO"] <- c(data[,"WHO_pre"], data[,"WHO_fu"])

data_gather[,"GAD"] <- c(data[,"GAD_pre"], data[,"GAD_fu"])

data_gather[,"Psychlops"] <- c(data[,"Psychlops_pre"], data[,"Psychlops_fu"])

#结局转化为2分类 PHQ GAD

data_gather <- data_gather%>%

mutate(., PHQ2 = ifelse(PHQ>=10,1,0))%>%

mutate(., GAD2 = ifelse(GAD>=10,1,0))

##modeling###

#少9

glmer_fit_PHQ[[j]] <- lmer(PHQ ~ Treatment+Time+Demog1+Demog11+(1|ID),

data =data_gather)

glmer_fit_WHO[[j]] <- lmer(WHO ~ Treatment+Time+Demog1+Demog11+(1|ID),

data =data_gather)

glmer_fit_GAD[[j]] <- lmer(GAD ~ Treatment+Time+Demog1+Demog11+(1|ID),

data =data_gather)

glmer_fit_Psychlops[[j]] <-lmer(Psychlops ~ Treatment+Time+Demog1+Demog11+(1|ID),

data =data_gather)

}

res_mi_long_mild <- list(summary(pool(glmer_fit_PHQ)),

summary(pool(glmer_fit_WHO)),

summary(pool(glmer_fit_GAD)),

summary(pool(glmer_fit_Psychlops)))

print(res_mi_long_mild)

#moderate####

mid_impu_data_moderate <- mid_impu_data%>%filter(.,PHQ_pre>=10&PHQ_pre<15)

data <- mid_impu_data_moderate

subset_index <- with(mid_impu_data,PHQ_pre>=10&PHQ_pre<15)

##将impute数据存入原数据集中

glmer_fit_PHQ <- list()

glmer_fit_WHO <- list()

glmer_fit_GAD <- list()

glmer_fit_Psychlops <- list()

#跑循环####

m=10

for (j in 1:m) { #10为m

for (i in 1:length(impu_var)) {

data[,impu_var[i]] <- impute(mid_impu_data[,impu_var[i]],

imp$imp[[impu_var[i]]][,j])[subset_index]

}

####获得gather data: pre & fu

data_gather <- gather(data,key=Time, value= PHQ, "PHQ_pre","PHQ_fu")

data_gather[,"WHO"] <- c(data[,"WHO_pre"], data[,"WHO_fu"])

data_gather[,"GAD"] <- c(data[,"GAD_pre"], data[,"GAD_fu"])

data_gather[,"Psychlops"] <- c(data[,"Psychlops_pre"], data[,"Psychlops_fu"])

#结局转化为2分类 PHQ GAD

data_gather <- data_gather%>%

mutate(., PHQ2 = ifelse(PHQ>=10,1,0))%>%

mutate(., GAD2 = ifelse(GAD>=10,1,0))

##modeling###

#少9和11

glmer_fit_PHQ[[j]] <- lmer(PHQ ~ Treatment+Time+Demog1+(1|ID),

data =data_gather)

glmer_fit_WHO[[j]] <- lmer(WHO ~ Treatment+Time+Demog1+(1|ID),

data =data_gather)

glmer_fit_GAD[[j]] <- lmer(GAD ~ Treatment+Time+Demog1+(1|ID),

data =data_gather)

glmer_fit_Psychlops[[j]] <-lmer(Psychlops ~ Treatment+Time+Demog1+(1|ID),

data =data_gather)

}

res_mi_long_moderate <- list(summary(pool(glmer_fit_PHQ)),

summary(pool(glmer_fit_WHO)),

summary(pool(glmer_fit_GAD)),

summary(pool(glmer_fit_Psychlops)))

print(res_mi_long_moderate)

#high####

mid_impu_data_high <- mid_impu_data%>%filter(.,PHQ_pre>=15)

data <- mid_impu_data_high

subset_index <- with(mid_impu_data,PHQ_pre>=15)

##将impute数据存入原数据集中

glmer_fit_PHQ <- list()

glmer_fit_WHO <- list()

glmer_fit_GAD <- list()

glmer_fit_Psychlops <- list()

#跑循环####

m=10

for (j in 1:m) { #10为m

for (i in 1:length(impu_var)) {

data[,impu_var[i]] <- impute(mid_impu_data[,impu_var[i]],

imp$imp[[impu_var[i]]][,j])[subset_index]

}

####获得gather data: pre & fu

data_gather <- gather(data,key=Time, value= PHQ, "PHQ_pre","PHQ_fu")

data_gather[,"WHO"] <- c(data[,"WHO_pre"], data[,"WHO_fu"])

data_gather[,"GAD"] <- c(data[,"GAD_pre"], data[,"GAD_fu"])

data_gather[,"Psychlops"] <- c(data[,"Psychlops_pre"], data[,"Psychlops_fu"])

#结局转化为2分类 PHQ GAD

data_gather <- data_gather%>%

mutate(., PHQ2 = ifelse(PHQ>=10,1,0))%>%

mutate(., GAD2 = ifelse(GAD>=10,1,0))

##modeling###

glmer_fit_PHQ[[j]] <- lmer(PHQ ~ Treatment+Time+Demog1+Demog9+Demog11+(1|ID),

data =data_gather)

glmer_fit_WHO[[j]] <-lmer(WHO ~ Treatment+Time+Demog1+Demog9+Demog11+(1|ID),

data =data_gather)

glmer_fit_GAD[[j]] <-lmer(GAD ~ Treatment+Time+Demog1+Demog9+Demog11+(1|ID),

data =data_gather)

glmer_fit_Psychlops[[j]] <-lmer(Psychlops ~ Treatment+Time+Demog1+Demog9+Demog11+(1|ID),

data =data_gather)

}

res_mi_long_high <- list(summary(pool(glmer_fit_PHQ)),

summary(pool(glmer_fit_WHO)),

summary(pool(glmer_fit_GAD)),

summary(pool(glmer_fit_Psychlops)))

# 5.LOCF ------------------------------------------------------------------

sbs_dat_basecom <- sbs_dat%>%filter(.,BaselineCompletion==1)

#5.1 结转####

#预后####

ana_dat_locf <- sbs_dat_basecom

ana_dat_locf$PHQ_post[is.na(ana_dat_locf$PHQ_post)] <-

with(ana_dat_locf[is.na(ana_dat_locf$PHQ_post),],

ifelse(is.na(PHQ_post_imput),PHQ_pre,PHQ_post_imput))

ana_dat_locf$WHO_post[is.na(ana_dat_locf$WHO_post)] <-

with(ana_dat_locf[is.na(ana_dat_locf$WHO_post),],

WHO_pre)

ana_dat_locf$GAD_post[is.na(ana_dat_locf$GAD_post)] <-

with(ana_dat_locf[is.na(ana_dat_locf$GAD_post),],

GAD_pre)

#Psychlops_pre先用均值填补

ana_dat_locf$Psychlops_pre <- impute(ana_dat_locf$Psychlops_pre,fun = mean)

ana_dat_locf$Psychlops_post[is.na(ana_dat_locf$Psychlops_post)] <-

with(ana_dat_locf[is.na(ana_dat_locf$Psychlops_post),],

Psychlops_pre)

#长期####

ana_dat_locf$PHQ_fu[is.na(ana_dat_locf$PHQ_fu)] <-

with(ana_dat_locf[is.na(ana_dat_locf$PHQ_fu),],

ifelse(is.na(PHQ_post_imput),PHQ_post,PHQ_post_imput))

ana_dat_locf$WHO_fu[is.na(ana_dat_locf$WHO_fu)] <-

with(ana_dat_locf[is.na(ana_dat_locf$WHO_fu),],

WHO_post)

ana_dat_locf$GAD_fu[is.na(ana_dat_locf$GAD_fu)] <-

with(ana_dat_locf[is.na(ana_dat_locf$GAD_fu),],

GAD_post)

ana_dat_locf$Psychlops_fu[is.na(ana_dat_locf$Psychlops_fu)] <-

with(ana_dat_locf[is.na(ana_dat_locf$Psychlops_fu),],

Psychlops_post)

#5.2 modeling####

#预后####

GLMM_pro_fun <- function(data){

data_gather <- gather(data,key=Time, value= PHQ, "PHQ_pre","PHQ_post")

data_gather[,"WHO"] <- c(data[,"WHO_pre"], data[,"WHO_post"])

data_gather[,"GAD"] <- c(data[,"GAD_pre"], data[,"GAD_post"])

data_gather[,"Psychlops"] <- c(data[,"Psychlops_pre"], data[,"Psychlops_post"])

#结局转化为2分类 PHQ GAD

data_gather <- data_gather%>%

mutate(., PHQ2 = ifelse(PHQ>=10,1,0))%>%

mutate(., GAD2 = ifelse(GAD>=10,1,0))

##modeling###

glmer_fit_PHQ <- lmer(PHQ ~ Treatment+Time+Demog1+Demog9+Demog11+(1|ID),

data =data_gather)

glmer_fit_WHO <- lmer(WHO ~ Treatment+Time+Demog1+Demog9+Demog11+(1|ID),

data =data_gather)

glmer_fit_GAD <- lmer(GAD ~ Treatment+Time+Demog1+Demog9+Demog11+(1|ID),

data =data_gather)

glmer_fit_Psychlops <-lmer(Psychlops ~ Treatment+Time+Demog1+Demog9+Demog11+(1|ID),

data =data_gather)

##用于整理数据

locf_summary_fun <- function(glmm.fit){

x <- coef(summary(glmm.fit))%>%as.data.frame()

colnames(x) <- c("estimate","std.error","df","statistic","p.value")

x

}

res_mi <- list(locf_summary_fun((glmer_fit_PHQ)),

locf_summary_fun((glmer_fit_WHO)),

locf_summary_fun((glmer_fit_GAD)),

locf_summary_fun((glmer_fit_Psychlops)))

print(res_mi)

}

res_locf_pro <- GLMM_pro_fun(ana_dat_locf)

#长期####

GLMM_pro_fun_long <- function(data){

data_gather <- gather(data,key=Time, value= PHQ, "PHQ_pre","PHQ_fu")

data_gather[,"WHO"] <- c(data[,"WHO_pre"], data[,"WHO_fu"])

data_gather[,"GAD"] <- c(data[,"GAD_pre"], data[,"GAD_fu"])

data_gather[,"Psychlops"] <- c(data[,"Psychlops_pre"], data[,"Psychlops_fu"])

#结局转化为2分类 PHQ GAD

data_gather <- data_gather%>%

mutate(., PHQ2 = ifelse(PHQ>=10,1,0))%>%

mutate(., GAD2 = ifelse(GAD>=10,1,0))

##modeling###

glmer_fit_PHQ_long <- lmer(PHQ ~ Treatment+Time+Demog1+Demog9+Demog11+(1|ID),

data =data_gather)

glmer_fit_WHO_long <- lmer(WHO ~ Treatment+Time+Demog1+Demog9+Demog11+(1|ID),

data =data_gather)

glmer_fit_GAD_long <- lmer(GAD ~ Treatment+Time+Demog1+Demog9+Demog11+(1|ID),

data =data_gather)

glmer_fit_Psychlops_long <-lmer(Psychlops ~ Treatment+Time+Demog1+Demog9+Demog11+(1|ID),

data =data_gather)

##用于整理数据

locf_summary_fun <- function(glmm.fit){

x <- coef(summary(glmm.fit)) %>% as.data.frame()

colnames(x) <- c("estimate","std.error","df","statistic","p.value")

x

}

res_mi_long <- list(locf_summary_fun((glmer_fit_PHQ_long)),

locf_summary_fun((glmer_fit_WHO_long)),

locf_summary_fun((glmer_fit_GAD_long)),

locf_summary_fun((glmer_fit_Psychlops_long)))

print(res_mi_long)

}

res_locf_long <- GLMM_pro_fun_long(ana_dat_locf)

COHENS_D(res_locf_long)

#5.3.1 subgroup for post####

mid_impu_data<- ana_dat_locf####

#female####

mid_impu_data_female <- mid_impu_data%>%filter(.,Demog1==2)

data <- mid_impu_data_female

data_gather <- gather(data,key=Time, value= PHQ, "PHQ_pre","PHQ_post")

data_gather[,"WHO"] <- c(data[,"WHO_pre"], data[,"WHO_post"])

data_gather[,"GAD"] <- c(data[,"GAD_pre"], data[,"GAD_post"])

data_gather[,"Psychlops"] <- c(data[,"Psychlops_pre"], data[,"Psychlops_post"])

#结局转化为2分类 PHQ GAD

data_gather <- data_gather%>%

mutate(., PHQ2 = ifelse(PHQ>=10,1,0))%>%

mutate(., GAD2 = ifelse(GAD>=10,1,0))

##modeling###

glmer_fit_PHQ <- lmer(PHQ ~ Treatment+Time+Demog11+(1|ID),

data =data_gather)

glmer_fit_WHO <- lmer(WHO ~ Treatment+Time+Demog11+(1|ID),

data =data_gather)

glmer_fit_GAD <- lmer(GAD ~ Treatment+Time+Demog11+(1|ID),

data =data_gather)

glmer_fit_Psychlops <-lmer(Psychlops ~ Treatment+Time+Demog11+(1|ID),

data =data_gather)

##用于整理数据

locf_summary_fun <- function(glmm.fit){

x <- coef(summary(glmm.fit))%>%as.data.frame()

colnames(x) <- c("estimate","std.error","df","statistic","p.value")

x

}

res_locf_pro_female <- list(locf_summary_fun((glmer_fit_PHQ)),

locf_summary_fun((glmer_fit_WHO)),

locf_summary_fun((glmer_fit_GAD)),

locf_summary_fun((glmer_fit_Psychlops)))

print(res_locf_pro_female)

#male####

mid_impu_data_male <- mid_impu_data%>%filter(.,Demog1==1)

data <- mid_impu_data_male

data_gather <- gather(data,key=Time, value= PHQ, "PHQ_pre","PHQ_post")

data_gather[,"WHO"] <- c(data[,"WHO_pre"], data[,"WHO_post"])

data_gather[,"GAD"] <- c(data[,"GAD_pre"], data[,"GAD_post"])

data_gather[,"Psychlops"] <- c(data[,"Psychlops_pre"], data[,"Psychlops_post"])

#结局转化为2分类 PHQ GAD

data_gather <- data_gather%>%

mutate(., PHQ2 = ifelse(PHQ>=10,1,0))%>%

mutate(., GAD2 = ifelse(GAD>=10,1,0))

##modeling###

glmer_fit_PHQ <- lmer(PHQ ~ Treatment+Time+Demog9+Demog11+(1|ID),

data =data_gather)

glmer_fit_WHO <- lmer(WHO ~ Treatment+Time+Demog9+Demog11+(1|ID),

data =data_gather)

glmer_fit_GAD <- lmer(GAD ~ Treatment+Time+Demog9+Demog11+(1|ID),

data =data_gather)

glmer_fit_Psychlops <-lmer(Psychlops ~ Treatment+Time+Demog9+Demog11+(1|ID),

data =data_gather)

res_locf_pro_male <- list(locf_summary_fun((glmer_fit_PHQ)),

locf_summary_fun((glmer_fit_WHO)),

locf_summary_fun((glmer_fit_GAD)),

locf_summary_fun((glmer_fit_Psychlops)))

print(res_locf_pro_male)

#mild####

mid_impu_data_mild <- mid_impu_data%>%filter(.,PHQ_pre>=5&PHQ_pre<10)

data <- mid_impu_data_mild

data_gather <- gather(data,key=Time, value= PHQ, "PHQ_pre","PHQ_post")

data_gather[,"WHO"] <- c(data[,"WHO_pre"], data[,"WHO_post"])

data_gather[,"GAD"] <- c(data[,"GAD_pre"], data[,"GAD_post"])

data_gather[,"Psychlops"] <- c(data[,"Psychlops_pre"], data[,"Psychlops_post"])

#结局转化为2分类 PHQ GAD

data_gather <- data_gather%>%

mutate(., PHQ2 = ifelse(PHQ>=10,1,0))%>%

mutate(., GAD2 = ifelse(GAD>=10,1,0))

##modeling###

glmer_fit_PHQ <- lmer(PHQ ~ Treatment+Time+Demog1+Demog11+(1|ID),

data =data_gather)

glmer_fit_WHO <- lmer(WHO ~ Treatment+Time+Demog1+Demog11+(1|ID),

data =data_gather)

glmer_fit_GAD <- lmer(GAD ~ Treatment+Time+Demog1+Demog11+(1|ID),

data =data_gather)

glmer_fit_Psychlops <-lmer(Psychlops ~ Treatment+Time+Demog1+Demog11+(1|ID),

data =data_gather)

res_locf_pro_mild <- list(locf_summary_fun((glmer_fit_PHQ)),

locf_summary_fun((glmer_fit_WHO)),

locf_summary_fun((glmer_fit_GAD)),

locf_summary_fun((glmer_fit_Psychlops)))

print(res_locf_pro_mild)

#moderate####

mid_impu_data_moderate <- mid_impu_data%>%filter(.,PHQ_pre>=10&PHQ_pre<15)

data <- mid_impu_data_moderate

data_gather <- gather(data,key=Time, value= PHQ, "PHQ_pre","PHQ_post")

data_gather[,"WHO"] <- c(data[,"WHO_pre"], data[,"WHO_post"])

data_gather[,"GAD"] <- c(data[,"GAD_pre"], data[,"GAD_post"])

data_gather[,"Psychlops"] <- c(data[,"Psychlops_pre"], data[,"Psychlops_post"])

#结局转化为2分类 PHQ GAD

data_gather <- data_gather%>%

mutate(., PHQ2 = ifelse(PHQ>=10,1,0))%>%

mutate(., GAD2 = ifelse(GAD>=10,1,0))

##modeling###

glmer_fit_PHQ <- lmer(PHQ ~ Treatment+Time+Demog1+(1|ID),

data =data_gather)

glmer_fit_WHO <- lmer(WHO ~ Treatment+Time+Demog1+(1|ID),

data =data_gather)

glmer_fit_GAD <- lmer(GAD ~ Treatment+Time+Demog1+(1|ID),

data =data_gather)

glmer_fit_Psychlops <-lmer(Psychlops ~ Treatment+Time+Demog1+(1|ID),

data =data_gather)

res_locf_pro_moderate <- list(locf_summary_fun((glmer_fit_PHQ)),

locf_summary_fun((glmer_fit_WHO)),

locf_summary_fun((glmer_fit_GAD)),

locf_summary_fun((glmer_fit_Psychlops)))

print(res_locf_pro_moderate)

#high####

mid_impu_data_high <- mid_impu_data%>%filter(.,PHQ_pre>=15)

data <- mid_impu_data_high

data_gather <- gather(data,key=Time, value= PHQ, "PHQ_pre","PHQ_post")

data_gather[,"WHO"] <- c(data[,"WHO_pre"], data[,"WHO_post"])

data_gather[,"GAD"] <- c(data[,"GAD_pre"], data[,"GAD_post"])

data_gather[,"Psychlops"] <- c(data[,"Psychlops_pre"], data[,"Psychlops_post"])

#结局转化为2分类 PHQ GAD

data_gather <- data_gather%>%

mutate(., PHQ2 = ifelse(PHQ>=10,1,0))%>%

mutate(., GAD2 = ifelse(GAD>=10,1,0))

##modeling###

glmer_fit_PHQ <- lmer(PHQ ~ Treatment+Time+Demog1+Demog9+Demog11+(1|ID),

data =data_gather)

glmer_fit_WHO <- lmer(WHO ~ Treatment+Time+Demog1+Demog9+Demog11+(1|ID),

data =data_gather)

glmer_fit_GAD <- lmer(GAD ~ Treatment+Time+Demog1+Demog9+Demog11+(1|ID),

data =data_gather)

glmer_fit_Psychlops <-lmer(Psychlops ~ Treatment+Time+Demog1+Demog9+Demog11+(1|ID),

data =data_gather)

res_locf_pro_high <- list(locf_summary_fun((glmer_fit_PHQ)),

locf_summary_fun((glmer_fit_WHO)),

locf_summary_fun((glmer_fit_GAD)),

locf_summary_fun((glmer_fit_Psychlops)))

print(res_locf_pro_high)

#5.3.2 subgroup for fu####

mid_impu_data<- ana_dat_locf####

#female####

mid_impu_data_female <- mid_impu_data%>%filter(.,Demog1==2)

data <- mid_impu_data_female

data_gather <- gather(data,key=Time, value= PHQ, "PHQ_pre","PHQ_fu")

data_gather[,"WHO"] <- c(data[,"WHO_pre"], data[,"WHO_fu"])

data_gather[,"GAD"] <- c(data[,"GAD_pre"], data[,"GAD_fu"])

data_gather[,"Psychlops"] <- c(data[,"Psychlops_pre"], data[,"Psychlops_fu"])

#结局转化为2分类 PHQ GAD

data_gather <- data_gather%>%

mutate(., PHQ2 = ifelse(PHQ>=10,1,0))%>%

mutate(., GAD2 = ifelse(GAD>=10,1,0))

##modeling###

glmer_fit_PHQ <- lmer(PHQ ~ Treatment+Time+Demog11+(1|ID),

data =data_gather)

glmer_fit_WHO <- lmer(WHO ~ Treatment+Time+Demog11+(1|ID),

data =data_gather)

glmer_fit_GAD <- lmer(GAD ~ Treatment+Time+Demog11+(1|ID),

data =data_gather)

glmer_fit_Psychlops <-lmer(Psychlops ~ Treatment+Time+Demog11+(1|ID),

data =data_gather)

##用于整理数据

locf_summary_fun <- function(glmm.fit){

x <- coef(summary(glmm.fit))%>%as.data.frame()

colnames(x) <- c("estimate","std.error","df","statistic","p.value")

x

}

res_locf_long_female <- list(locf_summary_fun((glmer_fit_PHQ)),

locf_summary_fun((glmer_fit_WHO)),

locf_summary_fun((glmer_fit_GAD)),

locf_summary_fun((glmer_fit_Psychlops)))

print(res_locf_long_female)

#male####

mid_impu_data_male <- mid_impu_data%>%filter(.,Demog1==1)

data <- mid_impu_data_male

data_gather <- gather(data,key=Time, value= PHQ, "PHQ_pre","PHQ_fu")

data_gather[,"WHO"] <- c(data[,"WHO_pre"], data[,"WHO_fu"])

data_gather[,"GAD"] <- c(data[,"GAD_pre"], data[,"GAD_fu"])

data_gather[,"Psychlops"] <- c(data[,"Psychlops_pre"], data[,"Psychlops_fu"])

#结局转化为2分类 PHQ GAD

data_gather <- data_gather%>%

mutate(., PHQ2 = ifelse(PHQ>=10,1,0))%>%

mutate(., GAD2 = ifelse(GAD>=10,1,0))

##modeling###

glmer_fit_PHQ <- lmer(PHQ ~ Treatment+Time+Demog9+Demog11+(1|ID),

data =data_gather)

glmer_fit_WHO <- lmer(WHO ~ Treatment+Time+Demog9+Demog11+(1|ID),

data =data_gather)

glmer_fit_GAD <- lmer(GAD ~ Treatment+Time+Demog9+Demog11+(1|ID),

data =data_gather)

glmer_fit_Psychlops <-lmer(Psychlops ~ Treatment+Time+Demog9+Demog11+(1|ID),

data =data_gather)

res_locf_long_male <- list(locf_summary_fun((glmer_fit_PHQ)),

locf_summary_fun((glmer_fit_WHO)),

locf_summary_fun((glmer_fit_GAD)),

locf_summary_fun((glmer_fit_Psychlops)))

print(res_locf_long_male)

#mild####

mid_impu_data_mild <- mid_impu_data%>%filter(.,PHQ_pre>=5&PHQ_pre<10)

data <- mid_impu_data_mild

data_gather <- gather(data,key=Time, value= PHQ, "PHQ_pre","PHQ_fu")

data_gather[,"WHO"] <- c(data[,"WHO_pre"], data[,"WHO_fu"])

data_gather[,"GAD"] <- c(data[,"GAD_pre"], data[,"GAD_fu"])

data_gather[,"Psychlops"] <- c(data[,"Psychlops_pre"], data[,"Psychlops_fu"])

#结局转化为2分类 PHQ GAD

data_gather <- data_gather%>%

mutate(., PHQ2 = ifelse(PHQ>=10,1,0))%>%

mutate(., GAD2 = ifelse(GAD>=10,1,0))

##modeling###

glmer_fit_PHQ <- lmer(PHQ ~ Treatment+Time+Demog1+Demog11+(1|ID),

data =data_gather)

glmer_fit_WHO <- lmer(WHO ~ Treatment+Time+Demog1+Demog11+(1|ID),

data =data_gather)

glmer_fit_GAD <- lmer(GAD ~ Treatment+Time+Demog1+Demog11+(1|ID),

data =data_gather)

glmer_fit_Psychlops <-lmer(Psychlops ~ Treatment+Time+Demog1+Demog11+(1|ID),

data =data_gather)

res_locf_long_mild <- list(locf_summary_fun((glmer_fit_PHQ)),

locf_summary_fun((glmer_fit_WHO)),

locf_summary_fun((glmer_fit_GAD)),

locf_summary_fun((glmer_fit_Psychlops)))

print(res_locf_long_mild)

#moderate####

mid_impu_data_moderate <- mid_impu_data%>%filter(.,PHQ_pre>=10&PHQ_pre<15)

data <- mid_impu_data_moderate

data_gather <- gather(data,key=Time, value= PHQ, "PHQ_pre","PHQ_fu")

data_gather[,"WHO"] <- c(data[,"WHO_pre"], data[,"WHO_fu"])

data_gather[,"GAD"] <- c(data[,"GAD_pre"], data[,"GAD_fu"])

data_gather[,"Psychlops"] <- c(data[,"Psychlops_pre"], data[,"Psychlops_fu"])

#结局转化为2分类 PHQ GAD

data_gather <- data_gather%>%

mutate(., PHQ2 = ifelse(PHQ>=10,1,0))%>%

mutate(., GAD2 = ifelse(GAD>=10,1,0))

##modeling###

glmer_fit_PHQ <- lmer(PHQ ~ Treatment+Time+Demog1+(1|ID),

data =data_gather)

glmer_fit_WHO <- lmer(WHO ~ Treatment+Time+Demog1+(1|ID),

data =data_gather)

glmer_fit_GAD <- lmer(GAD ~ Treatment+Time+Demog1+(1|ID),

data =data_gather)

glmer_fit_Psychlops <-lmer(Psychlops ~ Treatment+Time+Demog1+(1|ID),

data =data_gather)

res_locf_long_moderate <- list(locf_summary_fun((glmer_fit_PHQ)),

locf_summary_fun((glmer_fit_WHO)),

locf_summary_fun((glmer_fit_GAD)),

locf_summary_fun((glmer_fit_Psychlops)))

print(res_locf_long_moderate)

#high####

mid_impu_data_high <- mid_impu_data%>%filter(.,PHQ_pre>=15)

data <- mid_impu_data_high

data_gather <- gather(data,key=Time, value= PHQ, "PHQ_pre","PHQ_fu")

data_gather[,"WHO"] <- c(data[,"WHO_pre"], data[,"WHO_fu"])

data_gather[,"GAD"] <- c(data[,"GAD_pre"], data[,"GAD_fu"])

data_gather[,"Psychlops"] <- c(data[,"Psychlops_pre"], data[,"Psychlops_fu"])

#结局转化为2分类 PHQ GAD

data_gather <- data_gather%>%

mutate(., PHQ2 = ifelse(PHQ>=10,1,0))%>%

mutate(., GAD2 = ifelse(GAD>=10,1,0))

##modeling###

glmer_fit_PHQ <- lmer(PHQ ~ Treatment+Time+Demog1+Demog9+Demog11+(1|ID),

data =data_gather)

glmer_fit_WHO <- lmer(WHO ~ Treatment+Time+Demog1+Demog9+Demog11+(1|ID),

data =data_gather)

glmer_fit_GAD <- lmer(GAD ~ Treatment+Time+Demog1+Demog9+Demog11+(1|ID),

data =data_gather)

glmer_fit_Psychlops <-lmer(Psychlops ~ Treatment+Time+Demog1+Demog9+Demog11+(1|ID),

data =data_gather)

res_locf_long_high <- list(locf_summary_fun((glmer_fit_PHQ)),

locf_summary_fun((glmer_fit_WHO)),

locf_summary_fun((glmer_fit_GAD)),

locf_summary_fun((glmer_fit_Psychlops)))

print(res_locf_long_high)

# 6.0 绘制mice locf complete case 折线图 -------------------------------------------

#整理数据

#MICE

mean_MICE <- function(outcome){

X <- NULL

x_t1 <- NULL

x_t0 <- NULL

for (i in 1:10) {

X <- impute(imp$data[,outcome], imp$imp[[outcome]][,i])

x_t1 <- c(x_t1, X[imp$data$Treatment==1])

x_t0 <- c(x_t2, X[imp$data$Treatment==0])

}

cbind(c(mean(x_t1), mean(x_t0)),c(sd(x_t1),sd(x_t0)))

}

PHQ_chart_mice <- data.frame()

for (i in 1:3) {

PHQ_chart_mice <- rbind(PHQ_chart_mice,mean_MICE(impu_var[i]))

}

PHQ_chart_mice$time <- rep(c(0,2,5),each=2)

#locf

PHQ_chart_locf <- data.frame()

for (i in 1:3) {

PHQ_chart_locf <- rbind(PHQ_chart_locf,

cbind(c(mean(ana_dat_locf[ana_dat_locf$Treatment==1,impu_var[i]]),

mean(ana_dat_locf[ana_dat_locf$Treatment==0,impu_var[i]])),

c(sd(ana_dat_locf[ana_dat_locf$Treatment==1,impu_var[i]]),

sd(ana_dat_locf[ana_dat_locf$Treatment==0,impu_var[i]]))))

}

PHQ_chart_locf$time <- rep(c(0,2,5),each=2)

#raw

PHQ_chart_raw <- data.frame()

for (i in 1:3) {

PHQ_chart_raw <- rbind(PHQ_chart_raw,

cbind(c(mean(sbs_dat_basecom[sbs_dat_basecom$Treatment==1,impu_var[i]],na.rm=T),

mean(sbs_dat_basecom[sbs_dat_basecom$Treatment==0,impu_var[i]],na.rm=T)),

c(sd(sbs_dat_basecom[sbs_dat_basecom$Treatment==1,impu_var[i]],na.rm=T),

sd(sbs_dat_basecom[sbs_dat_basecom$Treatment==0,impu_var[i]],na.rm=T))))

}

PHQ_chart_raw$time <- rep(c(0,2,5),each=2)

PHQ_chart_data <- rbind(PHQ_chart_mice,PHQ_chart_locf,PHQ_chart_raw)

PHQ_chart_data$Treatment <- rep(c("ETAU","Step-by-Step"))

colnames(PHQ_chart_data)[1:2] <- c("mean", "sd")

PHQ_chart_data$condition <- rep(c("MICE","LOCF","raw data"),each=6)

PHQ_chart_data$time2 <- jitter(PHQ_chart_data$time)

p1 <- ggplot(PHQ_chart_data,

aes(x=time,y=mean,color=Treatment,shape=condition,linetype=condition))+

geom_point(size=3)+geom_line(position = position_dodge(0.1),cex=1)+

labs(title="")+theme(plot.title = element_text(hjust=0.5))+#标题

xlab("month since start of treatment")+ylab("PHQ-9 score")

p2 <- p1 +geom_errorbar(aes(ymin = mean - sd, ymax = mean + sd),

width = 0.25,cex=0.5)

p1

p2

ggsave("PHQ_chart_line.jpg", p1,width=8,height=4.5,dpi =300)

# ggsave("PHQ_chart_line.jpg", grid.arrange(p1,p2,ncol=1),width=10,height=6,dpi =300)

# #整理数据

# mean_MICE <- function(outcome){

# X <- NULL

# for (i in 1:m) {

# X <- c(X,impute(imp$data[,outcome], imp$imp[[outcome]][,i]))

# }

# X <- as.numeric(X)

# mean(X)

# }

#

# line_chart_data <- matrix(nrow=12,ncol=3)

# impu_var <- c("PHQ_pre","PHQ_post","PHQ_fu",

# "WHO_pre","WHO_post","WHO_fu",

# "GAD_pre","GAD_post","GAD_fu",

# "Psychlops_pre","Psychlops_post","Psychlops_fu")

# for (i in 1:length(impu_var)) {

# line_chart_data[i,] <- t(c(mean_MICE(impu_var[i]),

# mean(ana_dat_locf[,impu_var[i]]),

# mean(sbs_dat_basecom[,impu_var[i]],na.rm=T)))

# }

# colnames(line_chart_data) <- c("MICE","LOCF","raw data")

# line_chart_data <- as.data.frame(line_chart_data)

# line_chart_data$time <- rep(c(0,2,5),4)

# line_chart_data$indicator <- rep(c("PHQ","WHO","GAD","Psychlops"),each=3)

# line_chart_data <- line_chart_data%>%gather(.,key = "condition" , value = "outcome",MICE,LOCF,`raw data`)

#

# #绘图####

# p_PHQ<- ggplot(line_chart_data[line_chart_data$indicator=="PHQ",],

# aes(x=time,y=outcome,group=condition,color=condition,shape=condition))+

# geom_point(size=4)+geom_line(position = position_dodge(0.1),cex=1.3)+

# labs(title="PHQ")+theme(plot.title = element_text(hjust=0.5))#标题

#

# p_WHO<- ggplot(line_chart_data[line_chart_data$indicator=="WHO",],

# aes(x=time,y=outcome,group=condition,color=condition,shape=condition))+

# geom_point(size=4)+geom_line(position = position_dodge(0.1),cex=1.3)+

# labs(title="WHO")+theme(plot.title = element_text(hjust=0.5))#标题

#

# p_GAD<- ggplot(line_chart_data[line_chart_data$indicator=="GAD",],

# aes(x=time,y=outcome,group=condition,color=condition,shape=condition))+

# geom_point(size=4)+geom_line(position = position_dodge(0.1),cex=1.3)+

# labs(title="GAD")+theme(plot.title = element_text(hjust=0.5))#标题

#

# p_Psychlops<- ggplot(line_chart_data[line_chart_data$indicator=="Psychlops",],

# aes(x=time,y=outcome,group=condition,color=condition,shape=condition))+

# geom_point(size=4)+geom_line(position = position_dodge(0.1),cex=1.3)+

# labs(title="Psychlops")+theme(plot.title = element_text(hjust=0.5))#标题

#

# grid.arrange(p_PHQ, p_WHO, p_GAD,p_Psychlops, nrow=2)

# ggsave("折线图.jpg",grid.arrange(p_PHQ, p_WHO, p_GAD,p_Psychlops, nrow=2), width=8,height=8 )

# 6.sensitivity -----------------------------------------------------------

sbs_dat_all_comp <- sbs_dat%>%filter(.,Completion==1)

#6.1based on mice ####

ana_dat <- sbs_dat_all_comp

ana_dat_impu <- ana_dat%>%select(ID,Treatment,Demog1,Demog9,Demog11,

PHQ_pre,PHQ_post,PHQ_fu,

WHO_pre,WHO_post,WHO_fu,

GAD_pre,GAD_post,GAD_fu,

Psychlops_pre,Psychlops_post,Psychlops_fu,

Completion)

# colnames(ana_dat)

impu_var <- c("PHQ_pre","PHQ_post","PHQ_fu",

"WHO_pre","WHO_post","WHO_fu",

"GAD_pre","GAD_post","GAD_fu",

"Psychlops_pre","Psychlops_post","Psychlops_fu")

imp <- mice(ana_dat_impu,seed = 1234)

pred<- imp$predictorMatrix

# quick_pred<- quickpred(ana_dat_impu) #quick predictor 不好用

pred[setdiff(rownames(pred),impu_var),] <-0#不必填补的

pred[,impu_var[-c(1,4,7,10)]] <- 0 #predictor不需要post和fu

pred[,"Treatment"] <- 0

pred

imp <- mice(ana_dat_impu, predictorMatrix = pred, m=10,maxit = 20,seed = 1234,

printFlag = F,method = "pmm")

###计算Rhat

Rhat.mice(imp)

mid_impu_data <- imp$data

##写循环存结果

#预后效应####

res_mi_pro_all_comp <- GLMM_pro_fun(mid_impu_data)

#长期效应####

res_mi_long_all_comp <- GLMM_pro_fun_long(mid_impu_data)

#6.2based on locf ####

ana_dat <- sbs_dat_all_comp

#预后####

ana_dat_locf <- ana_dat

ana_dat_locf$PHQ_post[is.na(ana_dat_locf$PHQ_post)] <-

with(ana_dat_locf[is.na(ana_dat_locf$PHQ_post),],

ifelse(is.na(PHQ_post_imput),PHQ_pre,PHQ_post_imput))

ana_dat_locf$WHO_post[is.na(ana_dat_locf$WHO_post)] <-

with(ana_dat_locf[is.na(ana_dat_locf$WHO_post),],

WHO_pre)

ana_dat_locf$GAD_post[is.na(ana_dat_locf$GAD_post)] <-

with(ana_dat_locf[is.na(ana_dat_locf$GAD_post),],

GAD_pre)

#Psychlops_pre先用均值填补

ana_dat_locf$Psychlops_pre <- impute(ana_dat_locf$Psychlops_pre,fun = mean)

ana_dat_locf$Psychlops_post[is.na(ana_dat_locf$Psychlops_post)] <-

with(ana_dat_locf[is.na(ana_dat_locf$Psychlops_post),],

Psychlops_pre)

#长期####

ana_dat_locf$PHQ_fu[is.na(ana_dat_locf$PHQ_fu)] <-

with(ana_dat_locf[is.na(ana_dat_locf$PHQ_fu),],

ifelse(is.na(PHQ_post_imput),PHQ_post,PHQ_post_imput))

ana_dat_locf$WHO_fu[is.na(ana_dat_locf$WHO_fu)] <-

with(ana_dat_locf[is.na(ana_dat_locf$WHO_fu),],

WHO_post)

ana_dat_locf$GAD_fu[is.na(ana_dat_locf$GAD_fu)] <-

with(ana_dat_locf[is.na(ana_dat_locf$GAD_fu),],

GAD_post)

ana_dat_locf$Psychlops_fu[is.na(ana_dat_locf$Psychlops_fu)] <-

with(ana_dat_locf[is.na(ana_dat_locf$Psychlops_fu),],

Psychlops_post)

res_locf_pro_all_comp <- GLMM_pro_fun(ana_dat_locf)

res_locf_long_all_comp <- GLMM_pro_fun_long(ana_dat_locf)

# 7.completed case -------------------------------------------------------

##每个结局单独一个完整集

sbs_dat_noimpu_PHQ_post <- sbs_dat_basecom[!is.na(sbs_dat_basecom$PHQ_post),]

sbs_dat_noimpu_WHO_post <- sbs_dat_basecom[!is.na(sbs_dat_basecom$WHO_post),]

sbs_dat_noimpu_GAD_post <- sbs_dat_basecom[!is.na(sbs_dat_basecom$GAD_post),]

sbs_dat_noimpu_Psychlops_post <- sbs_dat_basecom[!is.na(sbs_dat_basecom$Psychlops_post),]

sbs_dat_noimpu_PHQ_fu <- sbs_dat_basecom[!is.na(sbs_dat_basecom$PHQ_fu),]

sbs_dat_noimpu_WHO_fu <- sbs_dat_basecom[!is.na(sbs_dat_basecom$WHO_fu),]

sbs_dat_noimpu_GAD_fu <- sbs_dat_basecom[!is.na(sbs_dat_basecom$GAD_fu),]

sbs_dat_noimpu_Psychlops_fu <- sbs_dat_basecom[!is.na(sbs_dat_basecom$Psychlops_fu),]

#7.0 比较PHQ缺失和完整数据集baseline####

sbs_dat_basecom_PHQ <- sbs_dat_basecom

sbs_dat_basecom_PHQ$PHQ_missing <- is.na(sbs_dat_basecom_PHQ$PHQ_post)

table_basecom_PHQ<- CreateTableOne(colnames(sbs_dat_basecom_PHQ)[-1],strata = "PHQ_missing",data = sbs_dat_basecom_PHQ,addOverall = T,includeNA = T)

myt1_basecom_PHQ <- print(table_basecom_PHQ,showAllLevels = T,missing = T)

write.csv(myt1_basecom_PHQ,"baseline_PHQ_missing.csv")

#7.1预后####

data_gather_fun <- function(data,select_index=T){

data <- data[select_index,]

data_gather <- gather(data,key=Time, value= PHQ, "PHQ_pre","PHQ_post")

data_gather[,"WHO"] <- c(data[,"WHO_pre"], data[,"WHO_post"])

data_gather[,"GAD"] <- c(data[,"GAD_pre"], data[,"GAD_post"])

data_gather[,"Psychlops"] <- c(data[,"Psychlops_pre"], data[,"Psychlops_post"])

#结局转化为2分类 PHQ GAD

data_gather <- data_gather%>%

mutate(., PHQ2 = ifelse(PHQ>=10,1,0))%>%

mutate(., GAD2 = ifelse(GAD>=10,1,0))

data_gather

}

##modeling###

glmer_fit_PHQ <- lmer(PHQ ~ Treatment+Time+Demog1+Demog9+Demog11+(1|ID),

data =data_gather_fun(sbs_dat_noimpu_PHQ_post))

glmer_fit_WHO <- lmer(WHO ~ Treatment+Time+Demog1+Demog9+Demog11+(1|ID),

data =data_gather_fun(sbs_dat_noimpu_WHO_post))

glmer_fit_GAD <- lmer(GAD ~ Treatment+Time+Demog1+Demog9+Demog11+(1|ID),

data =data_gather_fun(sbs_dat_noimpu_GAD_post))

glmer_fit_Psychlops <-lmer(Psychlops ~ Treatment+Time+Demog1+Demog9+Demog11+(1|ID),

data =data_gather_fun(sbs_dat_noimpu_Psychlops_post))

##用于整理数据

locf_summary_fun <- function(glmm.fit){

x <- coef(summary(glmm.fit))%>%as.data.frame()

colnames(x) <- c("estimate","std.error","df","statistic","p.value")

x

}

res_noimpu_pro <- list(locf_summary_fun((glmer_fit_PHQ)),

locf_summary_fun((glmer_fit_WHO)),

locf_summary_fun((glmer_fit_GAD)),

locf_summary_fun((glmer_fit_Psychlops)))

print(res_noimpu_pro)

#7.2长期####

data_gather_fun_long <- function(data){

data_gather <- gather(data,key=Time, value= PHQ, "PHQ_pre","PHQ_fu")

data_gather[,"WHO"] <- c(data[,"WHO_pre"], data[,"WHO_fu"])

data_gather[,"GAD"] <- c(data[,"GAD_pre"], data[,"GAD_fu"])

data_gather[,"Psychlops"] <- c(data[,"Psychlops_pre"], data[,"Psychlops_fu"])

#结局转化为2分类 PHQ GAD

data_gather <- data_gather%>%

mutate(., PHQ2 = ifelse(PHQ>=10,1,0))%>%

mutate(., GAD2 = ifelse(GAD>=10,1,0))

data_gather

}

##modeling###

glmer_fit_PHQ_long <- lmer(PHQ ~ Treatment+Time+Demog1+Demog9+Demog11+(1|ID),

data =data_gather_fun_long(sbs_dat_noimpu_PHQ_fu))

glmer_fit_WHO_long <- lmer(WHO ~ Treatment+Time+Demog1+Demog9+Demog11+(1|ID),

data =data_gather_fun_long(sbs_dat_noimpu_WHO_fu))

glmer_fit_GAD_long <- lmer(GAD ~ Treatment+Time+Demog1+Demog9+Demog11+(1|ID),

data =data_gather_fun_long(sbs_dat_noimpu_GAD_fu))

glmer_fit_Psychlops_long <-lmer(Psychlops ~ Treatment+Time+Demog1+Demog9+Demog11+(1|ID),

data =data_gather_fun_long(sbs_dat_noimpu_Psychlops_fu))

res_noimpu_long <- list(locf_summary_fun((glmer_fit_PHQ_long)),

locf_summary_fun((glmer_fit_WHO_long)),

locf_summary_fun((glmer_fit_GAD_long)),

locf_summary_fun((glmer_fit_Psychlops_long)))

print(res_noimpu_long)

#7.3.1 subgroup for post####

#female####

glmer_fit_PHQ <- lmer(PHQ ~ Treatment+Time+Demog1+Demog9+Demog11+(1|ID),

data = data_gather_fun(sbs_dat_noimpu_PHQ_post,select_index = sbs_dat_noimpu_PHQ_post$Demog1==2))

glmer_fit_WHO <- lmer(WHO ~ Treatment+Time+Demog1+Demog9+Demog11+(1|ID),

data =data_gather_fun(sbs_dat_noimpu_WHO_post,select_index = sbs_dat_noimpu_WHO_post$Demog1==2))

glmer_fit_GAD <- lmer(GAD ~ Treatment+Time+Demog1+Demog9+Demog11+(1|ID),

data =data_gather_fun(sbs_dat_noimpu_GAD_post,select_index = sbs_dat_noimpu_GAD_post$Demog1==2))

glmer_fit_Psychlops <-lmer(Psychlops ~ Treatment+Time+Demog1+Demog9+Demog11+(1|ID),

data =data_gather_fun(sbs_dat_noimpu_Psychlops_post,select_index = sbs_dat_noimpu_Psychlops_post$Demog1==2))

res_noimpu_pro_female <- list(locf_summary_fun((glmer_fit_PHQ)),

locf_summary_fun((glmer_fit_WHO)),

locf_summary_fun((glmer_fit_GAD)),

locf_summary_fun((glmer_fit_Psychlops)))

print(res_noimpu_pro_female)

#male####

glmer_fit_PHQ <- lmer(PHQ ~ Treatment+Time+Demog1+Demog9+Demog11+(1|ID),

data = data_gather_fun(sbs_dat_noimpu_PHQ_post,select_index = sbs_dat_noimpu_PHQ_post$Demog1==1))

glmer_fit_WHO <- lmer(WHO ~ Treatment+Time+Demog1+Demog9+Demog11+(1|ID),

data =data_gather_fun(sbs_dat_noimpu_WHO_post,select_index = sbs_dat_noimpu_WHO_post$Demog1==1))

glmer_fit_GAD <- lmer(GAD ~ Treatment+Time+Demog1+Demog9+Demog11+(1|ID),

data =data_gather_fun(sbs_dat_noimpu_GAD_post,select_index = sbs_dat_noimpu_GAD_post$Demog1==1))

glmer_fit_Psychlops <-lmer(Psychlops ~ Treatment+Time+Demog1+Demog9+Demog11+(1|ID),

data =data_gather_fun(sbs_dat_noimpu_Psychlops_post,select_index = sbs_dat_noimpu_Psychlops_post$Demog1==1))

res_noimpu_pro_male <- list(locf_summary_fun((glmer_fit_PHQ)),

locf_summary_fun((glmer_fit_WHO)),

locf_summary_fun((glmer_fit_GAD)),

locf_summary_fun((glmer_fit_Psychlops)))

print(res_noimpu_pro_male)

#mild####

#PHQ_pre>=5&PHQ_pre<10

glmer_fit_PHQ <- lmer(PHQ ~ Treatment+Time+Demog1+Demog9+Demog11+(1|ID),

data = data_gather_fun(sbs_dat_noimpu_PHQ_post,select_index = with(sbs_dat_noimpu_PHQ_post,PHQ_pre>=5&PHQ_pre<10)))

glmer_fit_WHO <- lmer(WHO ~ Treatment+Time+Demog1+Demog9+Demog11+(1|ID),

data =data_gather_fun(sbs_dat_noimpu_WHO_post,select_index =with(sbs_dat_noimpu_WHO_post,PHQ_pre>=5&PHQ_pre<10)))

glmer_fit_GAD <- lmer(GAD ~ Treatment+Time+Demog1+Demog9+Demog11+(1|ID),

data =data_gather_fun(sbs_dat_noimpu_GAD_post,select_index = with(sbs_dat_noimpu_GAD_post,PHQ_pre>=5&PHQ_pre<10)))

glmer_fit_Psychlops <-lmer(Psychlops ~ Treatment+Time+Demog1+Demog9+Demog11+(1|ID),

data =data_gather_fun(sbs_dat_noimpu_Psychlops_post,select_index = with(sbs_dat_noimpu_Psychlops_post,PHQ_pre>=5&PHQ_pre<10)))

res_noimpu_pro_mild <- list(locf_summary_fun((glmer_fit_PHQ)),

locf_summary_fun((glmer_fit_WHO)),

locf_summary_fun((glmer_fit_GAD)),

locf_summary_fun((glmer_fit_Psychlops)))

print(res_noimpu_pro_mild)

#moderate####

#PHQ_pre>=10&PHQ_pre<15

glmer_fit_PHQ <- lmer(PHQ ~ Treatment+Time+Demog1+Demog9+Demog11+(1|ID),

data = data_gather_fun(sbs_dat_noimpu_PHQ_post,select_index = with(sbs_dat_noimpu_PHQ_post,PHQ_pre>=10&PHQ_pre<15)))

glmer_fit_WHO <- lmer(WHO ~ Treatment+Time+Demog1+Demog9+Demog11+(1|ID),

data =data_gather_fun(sbs_dat_noimpu_WHO_post,select_index =with(sbs_dat_noimpu_WHO_post,PHQ_pre>=10&PHQ_pre<15)))

glmer_fit_GAD <- lmer(GAD ~ Treatment+Time+Demog1+Demog9+Demog11+(1|ID),

data =data_gather_fun(sbs_dat_noimpu_GAD_post,select_index = with(sbs_dat_noimpu_GAD_post,PHQ_pre>=10&PHQ_pre<15)))

glmer_fit_Psychlops <-lmer(Psychlops ~ Treatment+Time+Demog1+Demog9+Demog11+(1|ID),

data =data_gather_fun(sbs_dat_noimpu_Psychlops_post,select_index = with(sbs_dat_noimpu_Psychlops_post,PHQ_pre>=10&PHQ_pre<15)))

res_noimpu_pro_moderate <- list(locf_summary_fun((glmer_fit_PHQ)),

locf_summary_fun((glmer_fit_WHO)),

locf_summary_fun((glmer_fit_GAD)),

locf_summary_fun((glmer_fit_Psychlops)))

print(res_noimpu_pro_moderate)

#high####

#PHQ_pre>=15

glmer_fit_PHQ <- lmer(PHQ ~ Treatment+Time+Demog1+Demog9+Demog11+(1|ID),

data = data_gather_fun(sbs_dat_noimpu_PHQ_post,select_index = with(sbs_dat_noimpu_PHQ_post,PHQ_pre>=15)))

glmer_fit_WHO <- lmer(WHO ~ Treatment+Time+Demog1+Demog9+Demog11+(1|ID),

data =data_gather_fun(sbs_dat_noimpu_WHO_post,select_index =with(sbs_dat_noimpu_WHO_post,PHQ_pre>=15)))

glmer_fit_GAD <- lmer(GAD ~ Treatment+Time+Demog1+Demog9+Demog11+(1|ID),

data =data_gather_fun(sbs_dat_noimpu_GAD_post,select_index = with(sbs_dat_noimpu_GAD_post,PHQ_pre>=15)))

glmer_fit_Psychlops <-lmer(Psychlops ~ Treatment+Time+Demog1+Demog9+Demog11+(1|ID),

data =data_gather_fun(sbs_dat_noimpu_Psychlops_post,select_index = with(sbs_dat_noimpu_Psychlops_post,PHQ_pre>=15)))

res_noimpu_pro_high <- list(locf_summary_fun((glmer_fit_PHQ)),

locf_summary_fun((glmer_fit_WHO)),

locf_summary_fun((glmer_fit_GAD)),

locf_summary_fun((glmer_fit_Psychlops)))

print(res_noimpu_pro_high)

#7.3.2 subgroup for fu####

#female####

glmer_fit_PHQ <- lmer(PHQ ~ Treatment+Time+Demog1+Demog9+Demog11+(1|ID),

data = data_gather_fun(sbs_dat_noimpu_PHQ_fu,select_index = sbs_dat_noimpu_PHQ_fu$Demog1==2))

glmer_fit_WHO <- lmer(WHO ~ Treatment+Time+Demog1+Demog9+Demog11+(1|ID),

data =data_gather_fun(sbs_dat_noimpu_WHO_fu,select_index = sbs_dat_noimpu_WHO_fu$Demog1==2))

glmer_fit_GAD <- lmer(GAD ~ Treatment+Time+Demog1+Demog9+Demog11+(1|ID),

data =data_gather_fun(sbs_dat_noimpu_GAD_fu,select_index = sbs_dat_noimpu_GAD_fu$Demog1==2))

glmer_fit_Psychlops <-lmer(Psychlops ~ Treatment+Time+Demog1+Demog9+Demog11+(1|ID),

data =data_gather_fun(sbs_dat_noimpu_Psychlops_fu,select_index = sbs_dat_noimpu_Psychlops_fu$Demog1==2))

res_noimpu_long_female <- list(locf_summary_fun((glmer_fit_PHQ)),

locf_summary_fun((glmer_fit_WHO)),

locf_summary_fun((glmer_fit_GAD)),

locf_summary_fun((glmer_fit_Psychlops)))

print(res_noimpu_long_female)

#male####

glmer_fit_PHQ <- lmer(PHQ ~ Treatment+Time+Demog1+Demog9+Demog11+(1|ID),

data = data_gather_fun(sbs_dat_noimpu_PHQ_fu,select_index = sbs_dat_noimpu_PHQ_fu$Demog1==1))

glmer_fit_WHO <- lmer(WHO ~ Treatment+Time+Demog1+Demog9+Demog11+(1|ID),

data =data_gather_fun(sbs_dat_noimpu_WHO_fu,select_index = sbs_dat_noimpu_WHO_fu$Demog1==1))

glmer_fit_GAD <- lmer(GAD ~ Treatment+Time+Demog1+Demog9+Demog11+(1|ID),

data =data_gather_fun(sbs_dat_noimpu_GAD_fu,select_index = sbs_dat_noimpu_GAD_fu$Demog1==1))

glmer_fit_Psychlops <-lmer(Psychlops ~ Treatment+Time+Demog1+Demog9+Demog11+(1|ID),

data =data_gather_fun(sbs_dat_noimpu_Psychlops_fu,select_index = sbs_dat_noimpu_Psychlops_fu$Demog1==1))

res_noimpu_long_male <- list(locf_summary_fun((glmer_fit_PHQ)),

locf_summary_fun((glmer_fit_WHO)),

locf_summary_fun((glmer_fit_GAD)),

locf_summary_fun((glmer_fit_Psychlops)))

print(res_noimpu_long_male)

#mild####

#PHQ_pre>=5&PHQ_pre<10

glmer_fit_PHQ <- lmer(PHQ ~ Treatment+Time+Demog1+Demog9+Demog11+(1|ID),

data = data_gather_fun(sbs_dat_noimpu_PHQ_fu,select_index = with(sbs_dat_noimpu_PHQ_fu,PHQ_pre>=5&PHQ_pre<10)))

glmer_fit_WHO <- lmer(WHO ~ Treatment+Time+Demog1+Demog9+Demog11+(1|ID),

data =data_gather_fun(sbs_dat_noimpu_WHO_fu,select_index =with(sbs_dat_noimpu_WHO_fu,PHQ_pre>=5&PHQ_pre<10)))

glmer_fit_GAD <- lmer(GAD ~ Treatment+Time+Demog1+Demog9+Demog11+(1|ID),

data =data_gather_fun(sbs_dat_noimpu_GAD_fu,select_index = with(sbs_dat_noimpu_GAD_fu,PHQ_pre>=5&PHQ_pre<10)))

glmer_fit_Psychlops <-lmer(Psychlops ~ Treatment+Time+Demog1+Demog9+Demog11+(1|ID),

data =data_gather_fun(sbs_dat_noimpu_Psychlops_fu,select_index = with(sbs_dat_noimpu_Psychlops_fu,PHQ_pre>=5&PHQ_pre<10)))

res_noimpu_long_mild <- list(locf_summary_fun((glmer_fit_PHQ)),

locf_summary_fun((glmer_fit_WHO)),

locf_summary_fun((glmer_fit_GAD)),

locf_summary_fun((glmer_fit_Psychlops)))

print(res_noimpu_long_mild)

#moderate####

#PHQ_pre>=10&PHQ_pre<15

glmer_fit_PHQ <- lmer(PHQ ~ Treatment+Time+Demog1+Demog9+Demog11+(1|ID),

data = data_gather_fun(sbs_dat_noimpu_PHQ_fu,select_index = with(sbs_dat_noimpu_PHQ_fu,PHQ_pre>=10&PHQ_pre<15)))

glmer_fit_WHO <- lmer(WHO ~ Treatment+Time+Demog1+Demog9+Demog11+(1|ID),

data =data_gather_fun(sbs_dat_noimpu_WHO_fu,select_index =with(sbs_dat_noimpu_WHO_fu,PHQ_pre>=10&PHQ_pre<15)))

glmer_fit_GAD <- lmer(GAD ~ Treatment+Time+Demog1+Demog9+Demog11+(1|ID),

data =data_gather_fun(sbs_dat_noimpu_GAD_fu,select_index = with(sbs_dat_noimpu_GAD_fu,PHQ_pre>=10&PHQ_pre<15)))

glmer_fit_Psychlops <-lmer(Psychlops ~ Treatment+Time+Demog1+Demog9+Demog11+(1|ID),

data =data_gather_fun(sbs_dat_noimpu_Psychlops_fu,select_index = with(sbs_dat_noimpu_Psychlops_fu,PHQ_pre>=10&PHQ_pre<15)))

res_noimpu_long_moderate <- list(locf_summary_fun((glmer_fit_PHQ)),

locf_summary_fun((glmer_fit_WHO)),

locf_summary_fun((glmer_fit_GAD)),

locf_summary_fun((glmer_fit_Psychlops)))

print(res_noimpu_long_moderate)

#high####

#PHQ_pre>=15

glmer_fit_PHQ <- lmer(PHQ ~ Treatment+Time+Demog1+Demog9+Demog11+(1|ID),

data = data_gather_fun(sbs_dat_noimpu_PHQ_fu,select_index = with(sbs_dat_noimpu_PHQ_fu,PHQ_pre>=15)))

glmer_fit_WHO <- lmer(WHO ~ Treatment+Time+Demog1+Demog9+Demog11+(1|ID),

data =data_gather_fun(sbs_dat_noimpu_WHO_fu,select_index =with(sbs_dat_noimpu_WHO_fu,PHQ_pre>=15)))

glmer_fit_GAD <- lmer(GAD ~ Treatment+Time+Demog1+Demog9+Demog11+(1|ID),

data =data_gather_fun(sbs_dat_noimpu_GAD_fu,select_index = with(sbs_dat_noimpu_GAD_fu,PHQ_pre>=15)))

glmer_fit_Psychlops <-lmer(Psychlops ~ Treatment+Time+Demog1+Demog9+Demog11+(1|ID),

data =data_gather_fun(sbs_dat_noimpu_Psychlops_fu,select_index = with(sbs_dat_noimpu_Psychlops_fu,PHQ_pre>=15)))

res_noimpu_long_high <- list(locf_summary_fun((glmer_fit_PHQ)),

locf_summary_fun((glmer_fit_WHO)),

locf_summary_fun((glmer_fit_GAD)),

locf_summary_fun((glmer_fit_Psychlops)))

print(res_noimpu_long_high)

#7.4 sensitivity####

#预后####

#Completion==1

glmer_fit_PHQ <- lmer(PHQ ~ Treatment+Time+Demog1+Demog9+Demog11+(1|ID),

data = data_gather_fun(sbs_dat_noimpu_PHQ_post,select_index = with(sbs_dat_noimpu_PHQ_post,Completion==1)))

glmer_fit_WHO <- lmer(WHO ~ Treatment+Time+Demog1+Demog9+Demog11+(1|ID),

data =data_gather_fun(sbs_dat_noimpu_WHO_post,select_index =with(sbs_dat_noimpu_WHO_post,Completion==1)))

glmer_fit_GAD <- lmer(GAD ~ Treatment+Time+Demog1+Demog9+Demog11+(1|ID),

data =data_gather_fun(sbs_dat_noimpu_GAD_post,select_index = with(sbs_dat_noimpu_GAD_post,Completion==1)))

glmer_fit_Psychlops <-lmer(Psychlops ~ Treatment+Time+Demog1+Demog9+Demog11+(1|ID),

data =data_gather_fun(sbs_dat_noimpu_Psychlops_post,select_index = with(sbs_dat_noimpu_Psychlops_post,Completion==1)))

res_noimpu_pro_all_comp <- list(locf_summary_fun((glmer_fit_PHQ)),

locf_summary_fun((glmer_fit_WHO)),

locf_summary_fun((glmer_fit_GAD)),

locf_summary_fun((glmer_fit_Psychlops)))

print(res_noimpu_pro_all_comp)

#长期####

#Completion==1

glmer_fit_PHQ <- lmer(PHQ ~ Treatment+Time+Demog1+Demog9+Demog11+(1|ID),

data = data_gather_fun(sbs_dat_noimpu_PHQ_fu,select_index = with(sbs_dat_noimpu_PHQ_fu,Completion==1)))

glmer_fit_WHO <- lmer(WHO ~ Treatment+Time+Demog1+Demog9+Demog11+(1|ID),

data =data_gather_fun(sbs_dat_noimpu_WHO_fu,select_index =with(sbs_dat_noimpu_WHO_fu,Completion==1)))

glmer_fit_GAD <- lmer(GAD ~ Treatment+Time+Demog1+Demog9+Demog11+(1|ID),

data =data_gather_fun(sbs_dat_noimpu_GAD_fu,select_index = with(sbs_dat_noimpu_GAD_fu,Completion==1)))

glmer_fit_Psychlops <-lmer(Psychlops ~ Treatment+Time+Demog1+Demog9+Demog11+(1|ID),

data =data_gather_fun(sbs_dat_noimpu_Psychlops_fu,select_index = with(sbs_dat_noimpu_Psychlops_fu,Completion==1)))

res_noimpu_long_all_comp <- list(locf_summary_fun((glmer_fit_PHQ)),

locf_summary_fun((glmer_fit_WHO)),

locf_summary_fun((glmer_fit_GAD)),

locf_summary_fun((glmer_fit_Psychlops)))

print(res_noimpu_long_all_comp)

# 8.结果可视化 -------------------------------------------------------------------

##summary model

res_summary <- function(res){

res_summary <-data.frame()

for (i in 1:4) {

res_summary <-rbind(res_summary, res[[i]][2,])

}

res_summary$lci <- with(res_summary,estimate-1.96*std.error)

res_summary$uci <- with(res_summary,estimate+1.96*std.error)

res_summary <- round( select_if(res_summary, is.numeric),digits = 3)

res_summary$beta <- with(res_summary,paste(estimate, " (",lci,", ",uci,")",sep = ""))

res_summary$indicator <- c("PHQ","WHO","GAD", "Psychlops")

#与cohen'd merge

Cohens_summary <- COHENS_D(res)

res_summary <- inner_join(res_summary,Cohens_summary,by="indicator")

##排序

res_summary <- res_summary%>%select(indicator,beta,p.value,effectsize_cohens,effectsize_hedges)

#deparse(substitute提取对象名称

res_summary <- cbind(deparse(substitute(res)),res_summary)

colnames(res_summary)[1] <- "result_name"

print(res_summary)

}

RES_TABLE <- data.frame()

RES_TABLE <- rbind(RES_TABLE,

res_summary(res_mi_pro),

res_summary(res_mi_long),

res_summary(res_mi_pro_female),

res_summary(res_mi_pro_male),

res_summary(res_mi_pro_mild),

res_summary(res_mi_pro_moderate),

res_summary(res_mi_pro_high),

res_summary(res_mi_long_female),

res_summary(res_mi_long_male),

res_summary(res_mi_long_mild),

res_summary(res_mi_long_moderate),

res_summary(res_mi_long_high),

res_summary(res_mi_pro_all_comp),

res_summary(res_mi_long_all_comp),

res_summary(res_locf_pro),

res_summary(res_locf_long),

res_summary(res_locf_pro_female),

res_summary(res_locf_pro_male),

res_summary(res_locf_pro_mild),

res_summary(res_locf_pro_moderate),

res_summary(res_locf_pro_high),

res_summary(res_locf_long_female),

res_summary(res_locf_long_male),

res_summary(res_locf_long_mild),

res_summary(res_locf_long_moderate),

res_summary(res_locf_long_high),

res_summary(res_locf_pro_all_comp),

res_summary(res_locf_long_all_comp),

res_summary(res_noimpu_pro),

res_summary(res_noimpu_long),

res_summary(res_noimpu_pro_female),

res_summary(res_noimpu_pro_male),

res_summary(res_noimpu_pro_mild),

res_summary(res_noimpu_pro_moderate),

res_summary(res_noimpu_pro_high),

res_summary(res_noimpu_long_female),

res_summary(res_noimpu_long_male),

res_summary(res_noimpu_long_mild),

res_summary(res_noimpu_long_moderate),

res_summary(res_noimpu_long_high),

res_summary(res_noimpu_pro_all_comp),

res_summary(res_noimpu_long_all_comp)

)

write.csv(RES_TABLE,"model results 0927.csv")

save(imp,file="imp.rdata")

write.csv(ana_dat_locf,file = "locf.csv",row.names = F)

nrow(imp$data)
