# Supplementary material for: A WHO digital intervention to address depression among young Chinese adults: a type 1 effectiveness-implementation randomized controlled trial
Source: Transl Psychiatry. 2024 Feb 20;14:102. doi: 10.1038/s41398-024-02812-3 (PMC10879210; doi:10.1038/s41398-024-02812-3)
Supplement: Supplementary file 3 — Interview codebook [file 41398_2024_2812_MOESM3_ESM.docx]

| **Core Themes** | **Sub-themes** |
| --- | --- |
| **Reach** | **1A. Where did you get to know the program SbS? (Where）**  1. from the introduction by research staff  2. Email and WeChat – School news  3. Poster, leaflets on campus  **1B. What other places/platform would you suggest promoting the SbS ?　(Suggestion-channel)**  1. Events hold by local government departments or organizations (trade union, women union)  2. Online communities (WeChat group, Ins)  3. On campus activities, set up stalls, introduce by faculties/professionals  4. Referral from psycho clinics  5. WeChat mini app  6. Posters at school or bus stops  7. Work together with campus store for promotion  **1C. How do you think about the current advertisement? Do you think the advertisement is enough? (Feedback)**  1. Not adequate, could be ignored, no adequate information provided, can’t attract people with less interest or awareness of mental health  2. Adequate  **1D. What kind of advertisement would be useful to attract students like you to use the app? (Suggestion-Ads)**  1. Video ads (delivering stories, knowledge)  2. Highlight the compensations  3. Eye-catching, bigger posters or big screens  4. Introduction after simple psycho tests or mental health speech  5. More targeted information: what’s the target population, target conditions related to student’s life (anxiety, academic stress, procrastination); more positive terms, help set up expectation: potential benefits like what knowledge can be learned.  6. Introduce to more general population: help people identify their need for mental health service, increase awareness of self-mental health; introduce it as a service for the community. |
| **Adoption** | **2A. Did you encounter any problems? Or What stopped you finishing the assessment? (Assessment-quality)**  1. No problem  2. Inconvenient to skip items asking for private info  3. forget weekly assessment  4. CSQ-I items does not apply for control group  5. Hard to accurately recall items asking for frequency  **2B. What do you think about the length of the assessment? Appropriate? (Assessment-length)**    1. Acceptable  2. A bit long  **2C.** **Is the content easy/difficult to be understood? (Language)**  1. Easy to understand  2. Some phrases are based on Cantonese or Macao cultural context  3. One item in Psycholops was hard to understand  **2D. When did you usually use the app (e.g., during the day, evening; weekday, weekend; etc) and for how long? (Time to use)**  1. Night, after classes, before sleep  2. While walking on the way, on the bus  3. No fix time, when feel boring  4. Weekend  5. When feel distress  **2E. Did you use the features in SbS? How was it? (Use of exercise)**  1. Mood tracker (help monitor mood swings, use it as mood diary, upload pictures, allow edit within the day)  2. Read stories (relate to their life)  3. Behavior activation modules: Plan making/challenging activity (need more flexibility), social list  4. Grounding exercise and breath training (voice-guide)  5. Self-care modules: Grateful list, treat oneself better  6. My journey  **2F. Did you use any of these exercises/activities in your day-to-day life? If yes, which ones? How was it? (Daily use)**  1. Breathing practice is helpful (solve sleep problems, release anxious)  2. Plan making for challenge activities (for relaxation, use it as a diary, set up milestones for a mission)  3. Social list  4. Grateful list  5. Mood tracker  6. None |
| **Effectiveness** | **3A. How was your impression of SbS changed before and after installing/using it? (Impression)**    1. More content and more well-structured than expected, like a toolbox, rather than a list of knowledges or psycho test  2. Timesaving and concise  3. Relaxing  4. Lack of social network functions  5. Weekly contact unexpected  6. Take more time to unlock all the skills from app  7. Totally digitalized, no face-to-face interaction with real person, lack of engagement and motivation (severe case)  8. did not get rid of all negative affects  9. As a record of life  **3B. Did the program match with your idea? (Expectation)**  1. Basically match  2. Better than expectation  3. Less than expectation  4. No expectation  **3C. What have you learned from the program?/ What benefit did you receive from using SbS? (Benefits)**  1. More positive to find solutions for difficulties  2. Using skills and knowledges learned in the App to regulate mood, cope stress/anxiety, plan making, git rid of negative moods  3. Care more about oneself, better monitor self-mood  4. Awareness to seek for professional help  5. Communication with the E-helper  6. Real changes depend on self-effort, not by others or the app  7. Little is learned  **3D. On a scale from 1-10, 10 being the best, how satisfied were you with this program to help you to reduce your stress? (Satisfaction)**  1. 3- 5  2. 6-8  3. 9- 10 |
| **Implementation** | **4A. Did you encounter any problems when using SbS? If yes, how to solve? (Difficulty & Solution)**  1. Forget to use (interval too long, too much text typing, can be simplified by providing options, or voice record in activities like plan making to save time for users)  2. Automatic reminders are sometimes annoying (allow users to set up frequency of reminder)  3. Self-help, some behavior activation activities are difficult to practice (lack of supervision or motivation)  4. E-helper did not make weekly phone call  5. The stories will evoke unpleasant memory  6. Tech issue, sessions kept locked  7. No problem  **4B. Did you receive a call from e-helper? Did you pick it up? (Contact)**  1. Pickup several times or every time, or communicate via text  2. Weekly contact is too frequent (suggest twice a month)  3. Don’t know what to speak with E-helper  4. Only on-boarding call or follow-up call  5. Don’t want contact  **4C. What was your e-helper like? (E-helper-Impression)**  1. Nice people, good friend, patient  2. Nice voice, good mandarin  3. Feel good to talk with them, give positive feedbacks, good at summarizing their situations, trouble shooting  **4D. Were they helpful? (E-helper-Helpful)**  1. Helpful (exchange ideas, good listener, share secrets, not too official, technique support, teach skills, identify problems, remind and encourage using the App, review skills learned from the intervention)  2. Not very helpful, but good to talk with stranger |
| **Maintenance** | **5A. How could the intervention be improved for future use? (Suggestions)**  1. characters can be adapted (more comic-style, or animal)  2. Improve UI design (more introduction in main page, not appealing, too official, hard to find specific functions, make a clear list of functions on the main page)  3. More detailed guide (how to contact e-helper) and real-time feedbacks after weekly assessment or mood-tracker, more interaction with user (questions during stories)  4. Less storage need, mini program  5. More stories (more academic stress, bulling stress, adapt for mainland context)  6. Make the interval between sessions shorter  7. More reminders, change the delivery channel  8. Gamification (like add achievement features)  9. Add social media features (like the tree hole)  10. More customized content for different symptom severity, especially for mild symptoms, the current content describes more server cases  11. Improve the stability of the application  12. Organize events based on the app  13. Updating, text and video-based contents |
